# Polyethylene-like Polyesters: Strategies for Tailoring Mechanical Properties and Adhesion Performance

**DOI:** 10.1021/acs.macromol.5c01495

**Published:** 2025-08-22

**Authors:** Weronika Nowicka, Artur Rozanski, Farhan Ahmad Pasha, Lidia Jasinska-Walc, Rob Duchateau

**Affiliations:** † Department of Chemistry and Technology of Functional Materials, Chemical Faculty, 373502 Gdansk University of Technology, G. Narutowicza Str. 11/12, Gdansk 80-233, Poland; ‡ SABIC Technology & Innovation, Urmonderbaan 22, Geleen 6167 RD, Netherlands; § Centre of Molecular and Macromolecular Studies, 86897 Polish Academy of Sciences, Sienkiewicza 112, Lodz 90-363, Poland; ∥ SABIC Technology Center at KAUST, 25 Unity Blvd, Thuwal 23955, Saudi Arabia; ⊥ Chemical Product Engineering, Department of Chemical Engineering, University of Groningen, Nijenborgh 4, Groningen 9747 AG, Netherlands

## Abstract

Polycondensation of hydrogenated α,ω-dihydroxy polybutadiene soft blocks, hydrogenated α,ω-dihydroxy poly­(*cis*-cyclooctene) hard blocks, and succinic anhydride was utilized to synthesize high-density polyethylene-like (HDPE-like) and olefinic block copolymer-like (OBC-like) polyesters. The thus-prepared AABB-type long-spaced aliphatic polyesters (LSAPEs) exhibit tunable crystallinity, mechanical performance, and adhesion properties. Small- and wide-angle X-ray scattering (SAXS, WAXS) analyses revealed an increase in long-period values of the HDPE- and OBC-like polyesters from 25 to 36 nm and a gradual decrease in their crystallinity from 64% to 39% with increasing soft block content, resulting in lower density of the materials. Differential scanning calorimetry confirmed discrete block copolymer structures, as the melting temperature of the materials was preserved at the level of 118.8–127.8 °C, while the crystallinity degree decreased with increasing soft block content. Dynamic mechanical thermal analysis demonstrated that the viscoelastic properties of LSAPEs are comparable to those of commercial HDPE and OBCs. Comparing these AABB-type LSAPEs with recently reported AB-type analogues having lower molecular weight hard blocks and differently branched soft blocks revealed some interesting results. Furthermore, we investigated the adhesion of these polyolefin-like polyesters to aluminum and were able to support our findings with molecular dynamics simulations. The presence of only a limited number of ester functionalities and carboxylic chain-end groups provides a 20-fold higher adhesion to aluminum than the HDPE benchmark. This study demonstrates that polyolefin-like polyesters (LSAPEs) enable access to versatile materials revealing tunable mechanical and thermo-mechanical properties resembling those of various polyolefins, with significantly enhanced adhesion properties.

## Graphical abstract

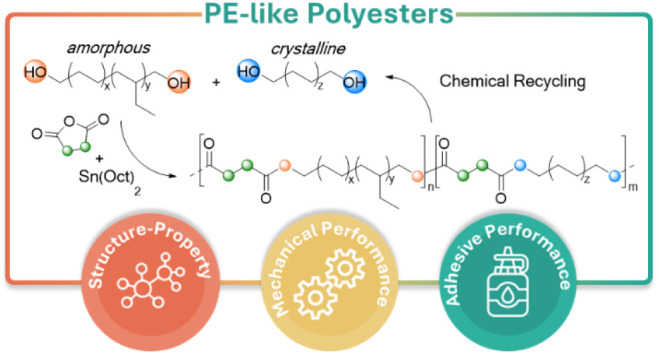

## Introduction

The undeniable industrial success and continuing popularity of polymers can be attributed to their excellent processability, wide range of properties, and cost-effectiveness compared to other materials.^1^ With applications ranging from packaging, textiles, and healthcare to construction materials and medical applications, polymers have monumentally improved the quality of our lives. With a market share of over 60%, polyolefins emerge as the most widely used synthetic polymers.^2,3^ Consequently, the global use of polyolefins, particularly in single-use packaging applications, contributes to large volumes of plastic waste and has resulted in a growing concern about plastic waste management and pollution.^4,5^ Although high-density polyethylene (HDPE) is one of the polymers having the highest recycling rates, most other polyolefin waste streams are less suitable for mechanical recycling. As an alternative, chemical recycling of polyolefins by means of pyrolysis is feasible but energy-intensive and—as a consequence of the lack of reactive bonds in the polymer chain—results in a wide range of products.^6,7,8^ It is well-recognized that future polymer systems should be designed adequately to ensure reusability and recyclability, to reduce the negative footprint of plastic linearity and move toward a circular polymer economy.^9,10,11,12,13^

Improved chemical recycling strategies for polyolefins have mainly focused on materials having weaker linkages in the main chain that can be selectively transformed into monomers^14,15,16,17,18^ or oligomeric building blocks^19,20,21,22,23^ and thus easily repolymerized. Typical functional groups susceptible to cleavage include carbon–carbon double bonds or heteroatom-containing (N, O, S) bonds that can be targeted by nucleophilic attack.^24,25^ For example, research related to polyethylene-like materials has focused on long-spaced aliphatic polyesters (LSAPEs), an abbreviation introduced by Mecking and coworkers.^26,27,28,29,30,31^ There are numerous synthetic protocols toward LSAPEs, which can be divided into two general categories: (a) ring-opening polymerization (ROP) of macrolactones^32,33,34,35^ and (b) step-growth polymerization of long-spaced α,ω-functionalized monomers. Both approaches introduce ester groups into the backbone of the polymer, thereby creating potentially degradable polyolefin-like materials. Polycondensation is a more versatile approach than ROP owing to the wider range of available macromonomers. The telechelic macromonomers used for the polycondensation are usually fatty acid-based C_12_–C_26_ diols, diesters, or diacids,^14,27,28,29,30,31,36^ or macromonomers obtained by (*i*) ring-opening or acyclic diene metathesis polymerization followed by hydrogenation,^26,37,38^ or (*ii*) coordinative chain transfer polymerization of regular olefins.^39^ The first report on recyclable LSAPEs produced by polycondensation was from Shiono et al., who performed the polycondensation of hydrogenated α,ω-dihydroxypolybutadiene with sebacoyl dichloride in the presence of pyridine.^40^ Mecking and coworkers have become synonymous with LSAPEs based on long-chain building blocks from renewable feedstock.^14,27,28,29,30,31^ More recently, Long and coworkers described the polycondensation of α,ω-dicarboxylic acid oligoethylenes derived from hydrogenated acid-functionalized polycyclooctene and 1,6-hexanediamine or 1,6-hexanediol to afford HDPE-like polyamides and polyesters, respectively.^38^ The production of such AABB polycondensates typically requires precise stoichiometric use of comonomers to yield sufficiently high molecular weight materials. To avoid this problem, Tang and coworkers have produced hydroxy-acid functionalized telechelic macromonomers by coordinative chain transfer polymerization, which can be converted into AB-type polyesters without the need of a second comonomer.^39^ Alternatively, Miyake and coworkers transformed α,ω-dihydroxyl functionalized telechelic macromonomers into AB-polyesters using a ruthenium-catalyzed dehydrogenation process.^19,21^ Although the above-mentioned strategies to produce AB-type polyesters are elegant, it would be desired to produce AABB-type polyesters from homofunctional telechelic macromonomers using a strategy where the stoichiometry of the comonomers is not affecting the final polyester’s molecular weight. Coates and coworkers approached this by producing α,ω-hydroxyethyl ester-functionalized telechelic macromonomers that can be polymerized in the same manner as polyethylene terephthalate.^22,41^

Here, we report the synthesis and characterization of a series of AABB-type polyolefin-resembling polyesters produced by the polycondensation of commercially available hydrogenated α,ω-dihydroxy polybutadiene, readily produced hydrogenated α,ω-dihydroxy poly­(*cis*-cyclooctene), and succinic anhydride. The use of succinic anhydride greatly facilitates the formation of high molecular weight polyesters in a short reaction time. The thus-obtained LSAPEs were designed to mimic both HDPE and OBCs in terms of their molecular and crystalline structures as well as thermomechanical properties. The polar functionalities in these polyesters not only allow chemical recycling by de- and repolymerization but also are expected to contribute to an increased adhesion to polar substrates, a highly desired feature that standard polyolefins lack.

## Results and Discussion

The target LSAPEs of this study were synthesized using two types of macromonomers: (*i*) hydrogenated α,ω-dihydroxy polybutadiene acting as a soft block that provides elasticity and tunable glass transition, and (*ii*) hydrogenated α,ω-dihydroxy poly­(*cis*-cyclooctene) as a hard block responsible for preserving high melting point and crystallinity. Telechelic oligoethylenes are well accessible via ROMP of *cis*-cyclooctenes followed by hydrogenation (for details, see Supporting Information). While this approach represents a straightforward and efficient synthetic protocol for hard blocks, preparation of soft blocks by this route is more challenging as it requires the use of alkyl-substituted cyclooctenes.^19,42^ Thus, as an alternative to soft blocks produced by ROMP, we decided to employ a hydrogenated hydroxyl-terminated polybutadiene resin, which as a commercial product is readily available and commonly used for coating and adhesive applications. To provide long spacing between ester linkages within the final material, the number-average molecular weights (*M̅*_n_) for the soft- and hard-block macrodiols are 2.0 and 2.9 kg/mol, respectively. While the highly crystalline hard block revealed a melting point of 129.9 °C, the soft block poly­(ethylene-*co*-butene), containing around 160 ethyl branches per 1000 carbons, is fully amorphous (Table 1 and Figure S1). Sn­(Oct)_2_-catalyzed melt polycondensation of a combination of these macrodiols and succinic anhydride provided a series of HDPE- and OBC-like polyesters. Whereas obtaining high molecular weight AABB polyesters—necessary to provide toughness and ductility—is often a challenge, the use of an excess of succinic anhydride as the polycondensation partner turned out to be a simple and highly effective solution to this issue (see Supporting Information for experimental details). The ability of the succinate chain ends to undergo back-biting, thereby releasing succinic anhydride, proved to be a highly efficient and simple route to ensure the right stoichiometry of reactive groups. ^1^H NMR spectroscopy confirmed successful incorporation of succinate units and the lack of residual unreacted succinic anhydride (Figures 1A, S2, and S3). As a result of the use of excess succinic anhydride, most polymer chains are succinic acid end-capped (Figure 1A). The thus obtained polyesters revealed satisfactory weight-average molecular weight (*M̅*_w_) ranging from 64.5 to 140.4 kg/mol with a polydispersity index between 2.5 and 3.2, as expected for step-growth polymerization (Table 1 and Figure 1B). The approach using succinic anhydride as a polycondensation partner allows high conversions and hence high molecular weight products in a considerably shorter reaction time as compared to alternative routes for polyolefin-like polyesters (Figures S4 and S5).^14,21,29^ Using this approach, a series of block copolymers, with a hard block content varying from 20 mol % (**PE-80/20**) to 80 mol % (**PE-20/80**) and a polyester consisting of only hard blocks (**PE-0/100**), with a comparable degree of polymerization (*X̅*_n_) were prepared. As the molecular weight of the soft block macromonomer is approximately 2/3 of that of the hard block macrodiol, the molecular weight of the obtained polyesters decreases with increasing soft block content for the same *X̅*_n_ (e.g., **PE-20/80** vs **PE-80/20**, Table 1).

**Table 1:** Characteristics of HDPE- and OBC-Like Polyesters and **HDPE**, **LLDPE**, and **OBC** Reference Samples

Sample	SB/HB^a^	*M̅*_w_ ^b^ (kg/mol)	*M̅*_n_ ^b^ (kg/mol)	*Đ*_M_ ^b^	*X̅*_n_ ^b^	SCB^c^/1000C	SCB*^b^* ^d^/1000C	*T*_m_ ^d^ (°C)	*T*_c_ ^d^ (°C)	Δ*H*_m_ ^d^ (J/g)	*T*_γ_ ^e^ (°C)	*T*_β_ ^e^ (°C)
**SB**	–	2.2	2.0	1.2	–	156	160	–	–	–	–	–
**HB**	–	5.4	2.9	1.9	–	0	0	129.9	115.3	253.0	–	–
**PE-0/100**	0/100	102.0	37.2	2.8	13	1	4	127.8	110.1	169.2	–108.2	–
**PE-20/80**	20/80	87.2	31.1	2.8	12	21	25	125.1	108.6	156.4	–116.4	–19.1
**PE-40/60**	40/60	140.4	43.3	3.2	17	48	55	121.1	105.8	136.3	–122.1	–28.3
**PE-60/40**	60/40	87.4	33.2	2.6	14	80	92	120.0	104.2	89.2	–127.3	–35.0
**PE-80/20**	80/20	64.8	26.4	2.5	12	118	138	118.8	99.0	56.4	–128.2	–36.8
**HDPE**	–	130.3	18.3	7.1	–	0	0	134.3	118.1	264.7	–107.2	–
**LLDPE**	–	97.0	37.0	2.6	–	16	15	112.0	92.6	99.6	–111.1	–
**OBC**	–	132.7	49.3	2.7	–	56	67	120.6	90.3	21.0	–122.7	–45.4

a Soft block/hard block ratio.

b Characterized by HT-SEC at 150 °C using oDCB as solvent.

c Determined with ^1^H NMR spectroscopy at 125 °C using TCE-D2 as solvent and BHT as antioxidant.

d Measured by DSC.

e Determined with ^1^H NMR spectroscopy at 125 °C using TCE-D_2_ as solvent and BHT as antioxidant.

**Figure 1:**
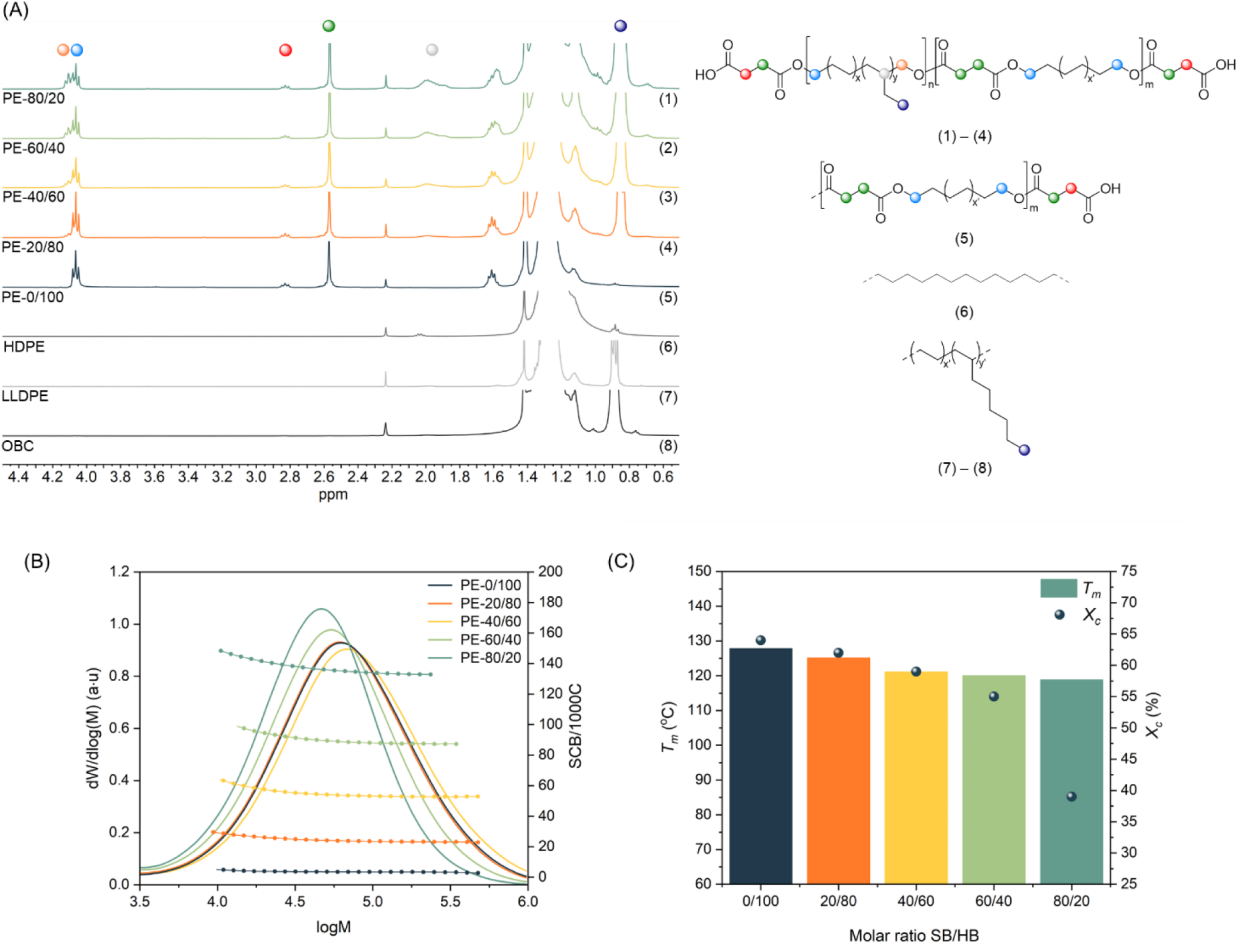
Molecular and thermal characterization of PE-like polyesters. Comparison of ^1^H NMR spectra (A), molecular weight distributions, average number of short-chain branching per 1000 carbons (SCB/1000C) (B), and melting temperature and level of crystallinity (C) for an HDPE-like polyester and a series of OBC-like polyesters.

However, the different number of branches might also affect the hydrodynamic volume and hence the measured (by high-temperature size exclusion chromatography, HT-SEC) molecular weight of the LSAPEs.^43,44^ Furthermore, the possibility of having ethyl branches close to the hydroxyl chain end group might hamper the polycondensation for LSAPEs rich in a branched soft block. The average number of branches per 1000 carbons in the block copolyesters as determined by HT-SEC is in good agreement with SCB/1000C values derived from ^1^H NMR spectroscopy (Table 1 and Figures 1B, S5) and increases gradually with increasing incorporation of the branched soft block (Figure S6). The entire series of LSAPEs displayed densities within the range observed for OBCs and HDPE, which is characteristic for lower-density polyethylenes (0.89 g/cm^3^ < *d* < 0.93 g/cm^3^) (Figure S7).

The HDPE- and all OBC-like polyesters exhibited high and well-defined melting transitions within the range 118.8–127.8 °C, as determined by differential scanning calorimetry (DSC) (Table 1 and Figure S8). **PE-0/100**, comprising only linear macromolecular building blocks, showed the highest *T*_m_ of 127.8 °C, as expected for the linear polyethylene analogue. As a comparison, the *T*_m_ of the corresponding linear macromonomer is 129.9 °C (Table 1). The near-linear correlation between the melting temperature and the number of ester groups in linear aliphatic polyesters reflects the penalty on the *T*_m_, following the Sanchez–Eby inclusion model, when ester groups are incorporated in the crystal lattice.^45,46,47^ Hence, the reduced melting point of **PE-0/100** in comparison with the **HDPE** benchmark (*T*_m_ = 134.3 °C) arises from the inclusion of ester groups in the crystalline phase, where the entire succinate unit most probably functions as a single defect in the crystal structure.^29,31^ In comparison, HDPE-like polymers synthesized via polycondensation of C_12_ to C_18_ macromonomers typically reveal a compromised melting point of a maximum of 99 °C due to high ester content,^14,16,29^ while polyesters based on high molecular weight telechelic building blocks (*M̅*_n_ > 8 kg/mol) show melting points above 130 °C, close to that of HDPE.^38,48^ Whereas the melting transitions for ethylene-based random copolymers are heavily affected by the amount of short-chain branching,^46,49^ for OBCs—comprising crystallizable and amorphous segments—the melting temperatures remain practically constant with increasing branching density, as the melting points are governed by the crystallinity of the hard blocks having few or no branches. Accordingly, the block copolymers **PE-80/20**–**PE-20/80** show rather constant melting points within the range 118.8–125.1 °C, which are in good agreement with the melting points of commercial grades of OBCs. The slight drop in *T*_m_ from **PE-20/80** to **PE-80/20** can be ascribed to the lower molecular weight of the soft block leading to a gradual increase in the succinate content in the block copolymers with increasing soft block content, giving the above-mentioned penalty on the melting temperature. Sample **PE-80/20**, representing the lowest *T*_m_ (118.8 °C) of all samples, still displayed a considerably higher melting point when compared with the selected **LLDPE** (*T*_m_ = 112.0 °C), despite the fact that the average number of branches (138/1000 C) for **PE-80/20** is considerably higher than that for **LLDPE** (15/1000 C) and the crystallinity level of **LLDPE** (*Χ*_c_ = 57%) is close to that of **PE-40/60** (*Χ*_c_ = 59%), which is significantly higher than that for **PE-80/20** (*Χ*_c_ = 39%; Figure 2). The melting enthalpy for the samples ranges from 169.2 J/g for the thermoplastic **PE-0/100** to 56.4 J/g for the highly elastomeric **PE-80/20**, whereas the difference in melting point is only 9 °C (Figure 1C).^50^ The melting enthalpy of the **OBC** reference sample is significantly lower compared to that of the softest **PE-80/20**, while having virtually the same *T*_m_. This can be explained by the fact that the hard blocks in the reference sample most likely contain some branches, whereas the hard blocks in the OBC-like polyesters are truly linear. Comparing **PE-0/100**–**PE-80/20** with similar OBC-like polyesters recently reported by Miyake and coworkers synthesized from the same hydrogenated poly­(cyclooctene) hard block but using poly­(3-hexylcyclooctene) instead of hydrogenated polybutadiene as the soft block provides interesting insight into the effect of the block copolymer structure on the thermal properties of these remarkable materials.^21^ Even though the molecular weights of both soft blocks (*M̅*_n_ ≈ 1.9 kg/mol) and of the final copolymers (average *M̅*_n_ ≈ 33 kg/mol) are comparable, the melting enthalpies and melting temperatures are significantly lower for the OBC-like polyester containing hydrogenated poly­(3-hexylcyclooctene) soft blocks than for their congener containing hydrogenated polybutadiene soft blocks (Figure S9).

**Figure 2:**
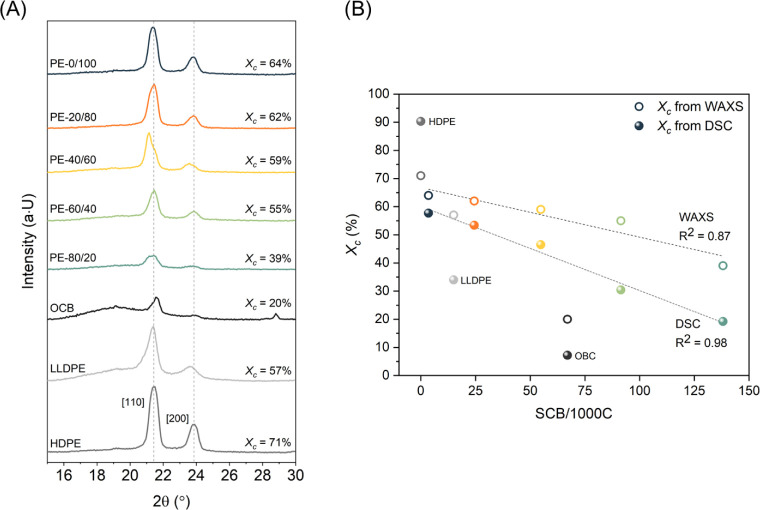
Crystalline structure of PE-like polyesters. WAXS profiles of PE-like polyesters and reference polyolefins. Weight percent crystallinity (*X*_c_) was estimated by deconvolution of the WAXS patterns (A). Correlation between the level of branching (HT-SEC SCB) and crystallinity of PE-like polyesters determined with WAXS and DSC. **HDPE**, **LLDPE**, and **OBC** are used as references (B).

Different factors might cause this difference, such as the difference in the molecular weight of the crystallizable hard block in **PE-0/100–PE-80/20** and Miyake’s OBC-like copolymers (*M̅*_n_ = 2.9 kg/mol vs *M̅*_n_ = 1.9 kg/mol, respectively), the difference in structure and polydispersity of the soft branched building blocks (i.e., hydrogenated polybutadiene and hydrogenated poly­(3-hexylcyclooctene)), and the type of linker (i.e., monoester vs succinate diester) used. Clearly, further investigation is required to get a better understanding of the influence of the macrodiols’ architecture on the thermal behavior of the LSAPEs.

The wide-angle X-ray scattering (WAXS) profiles of the LSAPEs (**PE-0/100–PE-80/20**) revealed the same peaks at scattering angles of 21.5° and 23.9°, representing the crystallographic [110] and [200] planes that are characteristic for an orthorhombic unit cell typical for polyethylene (Figure 2A).^30^ In line with the expectations, with an increasing soft block content, the intensity of the diffraction peaks diminishes, whereas the intensity of the amorphous halo becomes more pronounced. As a result, the volume degree of the crystallinity, estimated by deconvolution of the WAXS profiles, decreases gradually along the series (Figure 2). The crystallinity of **PE-0/100** (64%) is slightly lower than that of **HDPE** (71%), which can be explained by the penalty of introducing a succinate fragment in the crystalline phase. All other block copolymers show a gradual decrease of crystallinity with increasing content of soft blocks to a crystallinity level of 39% for the softest copolymer composition, **PE-80/20**, showing the inversely proportional correlation of the branching content (SCB/1000C) and the crystallinity level determined by both DSC and WAXS (Figure 2B), whereas such a correlation between the branching content and the melting temperature is absent. The differences in crystallinity degree estimated using WAXS and DSC might result from the 2θ degree range selected for the deconvolution method used for interpretation of the WAXS data. Additionally, such discrepancy might also arise from the amorphous halo partially covering crystalline regions. An increasing discrepancy between the *X*_c_ values calculated from DSC and WAXS for the polyesters containing a higher number of soft blocks clearly supports this conclusion.

The lamellar structure of the materials was analyzed by using small-angle X-ray scattering (SAXS). Figure S10 displays the SAXS profiles for both the LSAPEs and the reference polyolefins. The long period (LP), defined as the mean thickness of the crystalline and amorphous layers, was determined from the position of the q profile maximum (Table S1).^51,52,53^ Furthermore, based on the LP value and the degree of crystallinity (Figure 2), the thickness of the crystals for all analyzed materials was estimated (Table S1). The crystal thickness in **PE-0/100** (16.5 nm) was roughly 20% lower than that in the reference **HDPE** (20.2 nm), which supports the higher melting temperature of the latter sample (Table 1). With an increasing soft block content in the polyesters, a gradual decrease in the crystal thickness was observed (down to 14.2 nm for **PE-80/20**). It is worth noting that the observed changes in the microstructure of the crystalline component of **PE-0/100**–**PE-80/20** correlate well with the changes in their melting temperature (Tables 1 and S1). In the case of the **LLDPE** sample, which reveals thinner crystals (8.4 nm), its *T*_m_ was also significantly reduced (112.0 °C). The **OBC** reference sample exhibited the lowest crystal thickness (5.8 nm), yet its melting temperature remained relatively high (120.6 °C). This is likely due to the higher thermal resistance of the **OBC** crystals compared to those of **LLDPE** and the PE-like polyesters. Supporting evidence includes the shift of the scattering signal toward higher 2θ values (Figure 2), suggesting smaller interplanar distances in the **OBC** crystals.

To further understand the structure–property relationship of the OBC-like polyesters, the influence of the branching density on the viscoelastic properties and subambient temperature mechanical performance of the materials was elucidated using dynamic mechanical thermal analysis (DMTA) (Table 1 and Figures 3, S11). Starting from the low-temperature region, polyethylene and ethylene-based copolymers typically reveal three distinct mechanical relaxations, being γ, β, and α transitions.^54^ The maximum peak of the γ transition, typically affiliated with short-range motions of chains in the amorphous phase,^55^ starts from −128.2 °C for **PE-80/20** and gradually increases to −108.2 °C for the most crystalline **PE-0/100**. This gradual increase in *T*_γ_ with decreasing soft block content and thus lower branching content suggests that the short-range motions are hindered, likely due to the better molecular packing within the amorphous regions. The β transition, usually corresponding to the interfacial motion of chains, is often referred to as the glass transition temperature (*T*_g_) for branched polyethylenes such as LLDPE’s and OBC’s. The intensity of this transition amplifies with increasing branching density; therefore, **PE-80/20** displays the most pronounced relaxation at −36.8 °C. An increase in the content of the crystallizable linear hard block not only reduces the intensity of the β relaxation but also shifts the peak maximum toward −19.1 °C, as observed for **PE-20/80** (Figure S12). Linear **PE-0/100**, similarly to **HDPE**, lacks any β transition, and therefore *T*_γ_ is used as the primary glass transition temperature. The α relaxation—usually observed at elevated temperatures and attributed to mobility in the crystalline phase or at the crystal/amorphous interphase—was not investigated, as softening of the samples precluded precise determination of *T*_α_. Although we suspected that the linear **PE-0/100** would have physical and thermo-mechanical properties resembling **HDPE**, the tan δ profiles clearly indicate that this block copolymer possesses a *T*_g_ between that of **HDPE** and **LLDPE**. Polymers having between 20 and 80 mol % of soft blocks successfully mimic the low-temperature characteristics of LLDPEs and OBCs with varying branching content. Clearly, DMTA results showcased proper separation of hard and soft segments in the solid state for all block copolyesters as well as the tunability of the viscoelastic behavior of the materials simply by altering the ratio of hard and soft building blocks.

**Figure 3:**
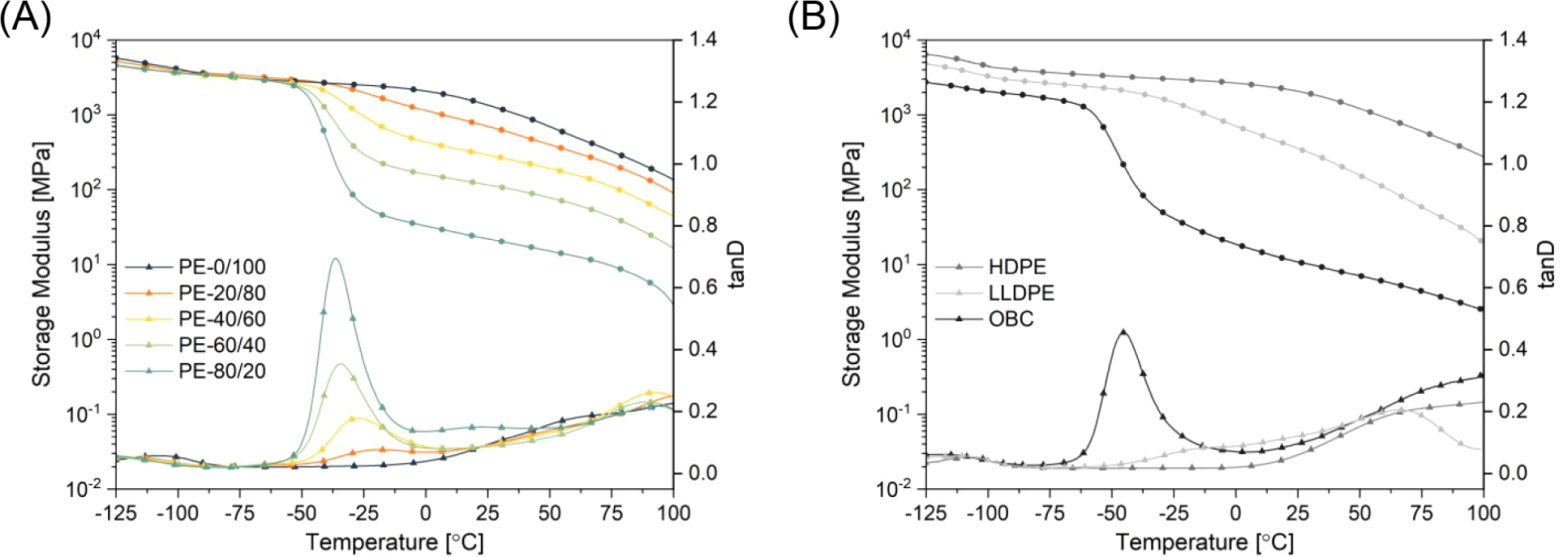
Viscoelastic properties and low-temperature performance of LSAPEs compared to commercially available polyolefins. Storage modulus and tanδ for LSAPEs (A) and reference polyolefins (B).

Uniaxial tensile tests were conducted to elucidate the mechanical performance of the synthesized LSAPEs (Table 2 and Figure 4A). The tensile properties of the block copolymers ranged from that of semicrystalline polyolefinic thermoplasts, as shown for **PE-0/100** and **PE-20/80**, to that of typical elastomers, as demonstrated by **PE-60/40** and **PE-80/20** (Figure S13). Sample **PE-0/100** exhibited a well-defined and localized yield point, plastic flow/strain-hardening stages, and a final elongation at break (1194%) that is higher than for the benchmark **HDPE** (922%) and surprisingly also higher than for the comparable HDPE-like polyester of Miyake and coworkers (800%).^21^ At the same time, the yield stress of **PE-0/100** (33 MPa) is considerably lower than for the **HDPE** benchmark (42 MPa), which can be attributed to the higher crystallinity/thickness of crystals of the latter sample, yet higher than that of the above-mentioned HDPE-like polyester reported by Miyake (25 MPa).^21^ The higher elongation at break and yield stress for **PE-0/100** as compared to the HDPE-like polyester reported by Miyake might originate from the higher molecular weight of the hard block in **PE-0/100**, leading to fewer crystal imperfections due to ester groups, and supports the hypothesis that the succinate moiety in these LSAPEs functions as a single crystal defect. The remarkable tensile strength and ductility of **PE-0/100** are reflected in an enhanced toughness (348 MJ/m^3^) in comparison to both **HDPE** (298 MJ/m^3^) and the HDPE-like polyester of Miyake and coworkers (150 MJ/m^3^; Figure 4B). Incorporation of soft segments in the copolymers results in the progressive decrease of the yield stress due to its dependency on the crystallinity level (Figure S13).^56^ Still, polyesters **PE-20/80** and **PE-40/60** displayed localized yield points (24 and 16 MPa, respectively), as well as strain hardening and an impressive elongation at break (985% and 918%, respectively). As expected for a multiblock copolymer structure comprising both elongation-prone soft segments and a crystalline domain that results in a thermoreversible cross-linked network, polyester **PE-60/40** revealed an exceptional elongation at break of 1300%. Such high extensibility of **PE-60/40** makes it not only competitive with **OBC** but also advantageous in terms of withstanding higher stress.^56^ For **PE-60/40** and **PE-80/20,** the elastomeric nature dominates the bulk properties, and hardly any or no yield point is observed in the stress–strain curve of **PE-60/40** and **PE-80/20**, respectively. For **PE-80/20**, containing the largest content of soft segments, the elongation at break (437%) and consequently the toughness (18 MJ/m^3^) were unexpectedly low and considerably lower than the corresponding OBC-like polyester of Miyake and coworkers having the same 80/20 soft/hard block composition (1000%). A likely cause is the combination of low crystallinity, lower ability of the highly branched soft blocks to undergo entanglements in combination with their lower molecular weight (as compared to the hard block, resulting in a lower copolymer molecular weight).^57^ Overall, the toughness of the LSAPEs can be tuned by simply varying the soft/hard block ratio, which is underlined by the near-linear correlation between *U*_T_ and the SCB/1000C (Figures S14 and S15). The investigated LSAPEs exhibited remarkable yield stress and toughness, higher than reported for thus far described HDPE- and OBC-like polyesters (Figure S16).^19,21^ Based on the limited data available, it is difficult to judge what causes this higher yield stress and toughness, as various differences in polymer structure (e.g., different molecular weight of the hard block, different branch type and branch density and polydispersity of the soft block, and different linker between the macrodiol blocks) of our and reported LSAPEs are likely to play a role.

**Table 2:** Mechanical and Adhesive Properties of PE-Like Polyesters and HDPE, LLDPE, and OBC References^a^

Sample	*σ*_y_ (MPa)	*σ*_b_ (MPa)	*ε*_b_ (%)	*U*_T_ (MJ/m^3^)	LSS Alu (MPa)	*W*_a_ (N/m)
PE-0/100	33.0 ± 0.7	33.5 ± 6.1	1194 ± 133	348 ± 53	8.1 ± 0.5	8679 ± 1483
PE-20/80	23.9 ± 0.6	32.3 ± 0.9	985 ± 52	224 ± 14	8.0 ± 0.7	9463 ± 2147
PE-40/60	15.5 ± 0.3	32.1 ± 2.3	918 ± 40	180 ± 18	7.2 ± 0.6	6581 ± 1277
PE-60/40	9.5 ± 0.1	12.4 ± 0.2	1300 ± 75	133 ± 10	7.4 ± 0.6	8076 ± 1558
PE-80/20	-	3.5 ± 0.3	437 ± 47	18 ± 3	4.5 ± 0.4	2751 ± 428
HDPE	41.8 ± 0.6	38.0 ± 6.1	922 ± 67	298 ± 31	0.4 ± 0.1	102 ± 29
LLDPE	13.4 ± 0.3	47.6 ± 2.3	1069 ± 58	298 ± 22	0.9 ± 0.1	193 ± 12
OBC	-	9.2 ± 0.8	1764 ± 90	68 ± 9	0.4 ± 0.1	102 ± 28

a *σ*_y_ – yield stress; *σ*_b_ – stress at break; *ε*_b_ – elongation at break; *U*_T_ – toughness; LSS – lap shear strength; *W*_a_ – work of adhesion.

**Figure 4:**
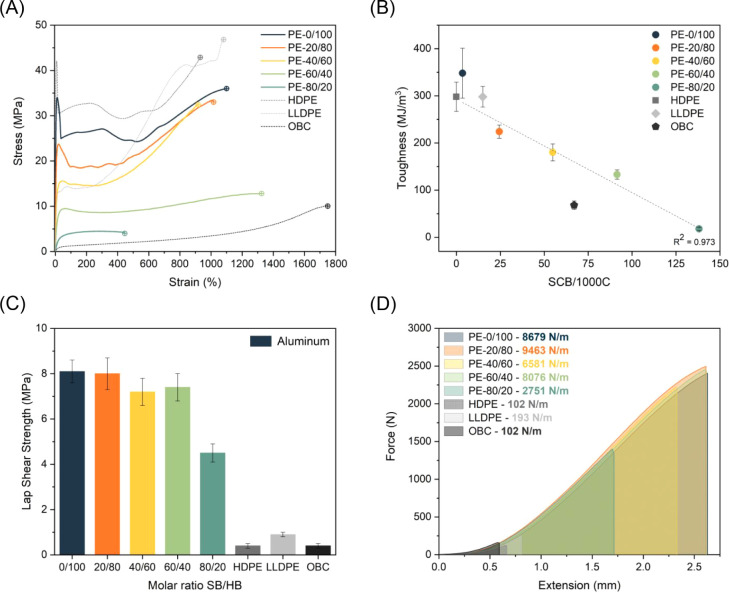
Mechanical and adhesive properties. Representative stress–strain curves of PE-like polyesters and polyolefins used as benchmarks (A); correlation between the level of branching (HT-SEC SCB) and toughness of PE-like polyesters and polyolefins used as benchmarks (B); adhesive performance of PE-like polyesters and reference polyolefins in bonding aluminum (C); graphic representation of the work of adhesion of PE-like polyesters and reference polyolefins (D).

It was argued that the ester functionalities in the backbone of the copolymers as well as the chain-end carboxyl functionalities could contribute to an enhanced adhesion of these copolymers to polar substrates compared to conventional polyolefins that lack polar groups. Indeed, hydroxyl-functionalized propylene copolymers have demonstrated exceptionally strong adhesion to, for example, metals like aluminum and steel, despite a very low functionality level in an otherwise highly apolar polymer.^58^ Recently, Tang and coworkers have shown that polyolefin-like AB-type polyesters adhere significantly stronger to PEEK and PET as compared to LLDPE and OBC benchmark samples.^39^ Likewise, Zhao et al. demonstrated a significantly improved adhesion of their OBC-like polyesters to aluminum.^19^ To quantitatively assess the adhesion of our LSAPEs to aluminum surfaces, lap shear strength (LSS) and work of adhesion (*W*_a_) were determined (Table 2 and Figure 4C). Of all tested samples, **PE-0/100** revealed an outstanding adhesion strength of 8.1 MPa, 20-fold higher than the adhesive strength of the **HDPE** benchmark (0.4 MPa), while the work required to disconnect the aluminum surface bonded with **PE-0/100** is close to 2 orders of magnitude higher in comparison to the **HDPE** (Table 2 and Figure 4D). High adhesion strength is retained throughout almost the whole series of polyesters up to **PE-60/40** (LLS: 7.2–8.1 MPa; *W*_a_: 6581–9463 N/m) and outperforms **LLDPE** and **OBC** (LLS: 0.4–0.9 MPa; *W*_a_: 102–193 N/m) both in shear strength and work. In comparison with the more crystalline polyesters, the least crystalline **PE-80/20** demonstrated a somewhat reduced, but nevertheless still over 10 times higher adhesion strength than the corresponding **OBC** benchmark sample (4.5 MPa vs 0.4 MPa). Knowing that an increased crystallinity level provides a robust crystalline thermo-reversible cross-linked network that can resist high deformations during the mechanical testing, it is likely that the diminished crystallinity in combination with the somewhat lower molecular weight of **PE-80/20** plays a role.^59^ However, increasing branching content from the soft block might also impede the adhesion due to enhanced creep, as is observed for more elastomeric OBCs.

Hence, to elucidate whether the ester functionalities migrate to the polar surface of the aluminum specimen and to investigate the influence of the branches while increasing the soft block content on the adhesive performance, molecular dynamics simulations (MD) were performed.^60,61,62^ The energy of adhesion (*E*_adh_)—a measure for the affinity to the alumina oxide surface—was determined for three representative LSAPEs, **PE-0/100**, **PE-40/60**, and **PE-80/20**, varying in the degree of branching (Figure 5 and Table S2). The **PE-0/100** model, with a theoretical *M̅*_*n*(MD PE‑0/100)_ of 38.7 kg/mol, demonstrated the most negative *E*_adh_ (−1922 kcal/mol), indicating that the linear polyester reveals the highest affinity to interact with the alumina surface, which agrees with the observed high adhesion for **PE-0/100** to aluminum. For the **PE-40/60** and **PE-80/20** models, representing polyesters with a higher soft block content (*M*_*n*(MD PE‑40/60)_ = 43.7 kg/mol and *M*_*n*(MD PE‑80/20)_ = 27.0 kg/mol), the *E*_adh_ values gradually increase but remain negative regardless of the high degree of branching (−1725 and −1180 kcal/mol, respectively). Such decreasing trend with increasing branching content most presumably derives from two factors: (*i*) disturbed lamellar packing close to the alumina oxide surface, and (*ii*) the number of oxygen atoms participating in bonding to the aluminum oxide surface. The **PE-0/100** model displays a regular and dense lamellar packing of approximately 3.7 Å with 16 oxygen atoms near the aluminum surface. This clearly demonstrates the tendency of oxygens to migrate to the alumina surface. Conversely, the **PE-40/60** model reveals a less regular lamellar packing containing 12 oxygens at the interface, and the **PE-80/20** model exhibits the most disturbed lamellar packing with only nine oxygen atoms participating in bonding to the aluminum oxide (Figure 5B). Increasing disturbance of the lamellar packing, independently confirmed by SAXS, translates to an increase in entropy, which for the most branched polymer results in a smaller number of ester groups participating in bonding to the surface. For randomly hydroxyl-functionalized propylene copolymers, it is known that, although the absolute contribution of the sum of the polar interactions is just a small fraction of the observed adhesive strength, an increase in these interactions has a profound effect on the overall adhesive strength.^63^ We assume that the same is true for these LSAPEs and support the observation that the most regular polymer (**PE-0/100**), having the most ester functionalities interacting with the alumina surface, shows the highest adhesive strength.

**Figure 5:**
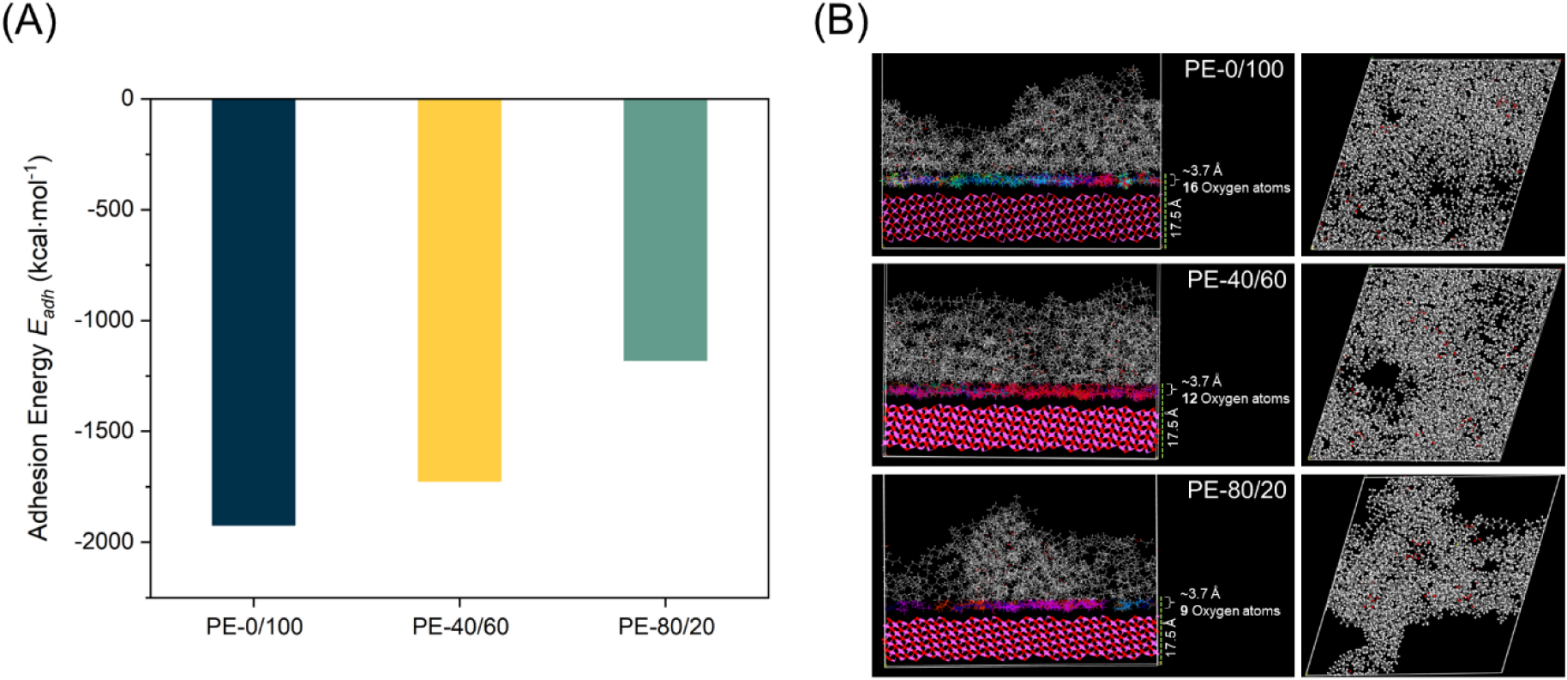
Molecular dynamics simulations. Adhesion energy (*E*_adh_) of the representative PE-0/100, PE-40/60, and PE-80/20 (A). Molecular dynamics simulation of polymer adhesion on an aluminum oxide (Al_2_O_3_) surface and facets, where red spheres correspond to ester groups (B).

## Conclusions

A series of high molecular weight HDPE-like and OBC-like polyesters was successfully synthesized using melt polycondensation of succinic anhydride, hydrogenated α,ω-dihydroxy polybutadiene soft blocks, and hydrogenated α,ω-dihydroxy poly­(*cis*-cyclooctene) hard blocks. The ability of succinate end groups to undergo a back-biting process, releasing succinic anhydride, proved to be a highly efficient and simple route to ensure the right stoichiometry of reactive groups, resulting in high molecular weight AABB-type polyesters. The comparable penalty on the melting temperature of the ester groups in these AABB-type polyesters and analogous AB-type LSAPEs indicates that the succinate group gives a similar defect in the crystal lattice as a single ester group. The inverse proportional correlation between the branching content (i.e., soft block content) and the crystallinity, as well as the absence of such a correlation between the branching content and the melting point, being characteristic for multiblock copolymers, indicates that the melting temperature is governed by the crystallinity of the hard block. Consequently, the viscoelastic behavior and tensile properties of the OBC-like polyesters can be tuned by simply adjusting the ratio of soft and hard blocks. Interestingly, no clear difference could be observed between the thermal and mechanical properties of multiblock copolymers containing either amorphous hydrogenated polybutadiene or hydrogenated poly­(3-hexylcyclooctene) blocks, although it might have been expected that the difference in branching density (approximately 2× higher for the hydrogenated polybutadiene) and the difference in branching length (C2 vs C6) of these soft blocks might influence the mechanical and tensile properties of the materials. The presence of ester functionalities in the polymer’s backbone and carboxylic acid end groups provides remarkable adhesion to polar surfaces like aluminum compared to conventional polyolefins. Here, the branched soft block plays a crucial role in tuning the adhesive strength, as an increasing soft block content results in an increased disturbance of the lamellar packing, which results in fewer ester groups being available for interacting with the polar substrate surface and hence a lower adhesive strength. The strong interfacial bonding of the LSAPEs provides potential application in coatings, adhesives, and packaging.

## Supplementary Material



## Notes

Complete experimental section including general considerations, analytical techniques, HT-SEC spectra, thermograms, and related calculations (PDF)

## Abbreviations

HB: hard block
SB: soft block
ROMP: ring-opening metathesis polymerization
HDPE: high-density polyethylene
LLDPE: linear low-density polyethylene
OBC: olefin block copolymer
DSC: differential scanning calorimetry
HT-SEC: high-temperature size exclusion chromatography
NMR: nuclear magnetic resonance
DMTA: dynamic mechanical thermal analysis
SAXS: small-angle X-ray scattering
WAXS: wide-angle X-ray scattering
PEEK: polyether ether ketone
PET: polyethylene terephthalate
MD: molecular dynamics

## References

[1] Geyer R., Jambeck J. R., Law K. L. (2017). Production, use, and fate of all
plastics ever made. Sci. Adv..

[2] Jambeck J. R., Geyer R., Wilcox C., Siegler T. R., Perryman M., Andrady A., Narayan R., Law K. L. (2015). Plastic
waste inputs
from land into the ocean. Science.

[3] Plastics The Fast Facts 2023; PlasticsEurope, 2023. https://plasticseurope.org/knowledge-hub/plastics-the-fast-facts-2023/.

[4] Hauschild M. Z., Bjørn A. (2023). Pathways to
sustainable plastics. Nat. Sustain..

[5] Hoornweg D., Bhada-Tata P., Kennedy C. (2013). Environment: Waste production must
peak this century. Nature.

[6] Walendziewski J. (2006). Thermal and
Catalytic Conversion of Polyolefins. Feedstock
Recycl. Pyrolysis Waste Plast..

[7] Lee K.-H. (2006). Thermal
and Catalytic Degradation of Waste HDPE. Feedstock
Recycl. Pyrolysis Waste Plast..

[8] Hees T., Zhong F., Stürzel M., Mülhaupt R. (2019). Tailoring
Hydrocarbon Polymers and All-Hydrocarbon Composites for Circular Economy. Macromol. Rapid Commun..

[9] Kakadellis S., Rosetto G. (2021). Achieving a circular bioeconomy for plastics. Science.

[10] Uekert T., Singh A., DesVeaux J. S., Ghosh T., Bhatt A., Yadav G., Afzal S., Walzberg J., Knauer K. M., Nicholson S. R., Beckham T. G., Carpenter A. C. (2023). Technical
Economic, and Environmental Comparison of Closed-Loop Recycling Technologies
for Common Plastics. ACS Sustainable Chem. Eng..

[11] Coates G. W., Getzler Y. D. Y. L. (2020). Chemical recycling to monomer for an ideal, circular
polymer economy. Nat. Rev. Mater..

[12] Aarsen C. V., Liguori A., Mattsson R., Sipponen M. H., Hakkarainen M. (2024). Designed to
Degrade: Tailoring Polyesters for Circularity. Chem. Rev..

[13] Haque F. M., Ishibashi J. S. A., Lidston C. A. L., Shao H., Bates F. S., Chang A. B., Coates G. W., Cramer C. J., Dauenhauer P. J., Dichtel W. R., Ellison C. J., Gormong E. A., Hamachi L. S., Hoye T. R., Jin M., Kalow J. A., Kim H. J., Kumar G., La Salle C. J., Liffland S., Lipinski B. M., Pang Y., Parveen R., Peng X., Popowski Y., Prebihalo E. A., Reddi Y., Reineke T. M., Sheppard D. T., Swartz J. L., Tolman W. B., Vlaisavljevich B., Wissinger J., Xu S., Hillmyer M. A. (2022). Defining the Macromolecules
of Tomorrow through Synergistic Sustainable Polymer Research. Chem. Rev..

[14] Häußler M., Eck M., Rothauer D., Mecking S. (2021). Closed-loop recycling of polyethylene-like
materials. Nature.

[15] Li X.-L., Clarke R. W., An H.-Y., Gowda R. R., Jiang J.-Y., Xu T.-Q., Chen E. Y.-X. (2023). Dual
Recycling of Depolymerization
Catalyst and Biodegradable Polyester that Markedly Outperforms Polyolefins. Angew. Chem., Int. Ed..

[16] Nelson T. F., Rothauer D., Sander M., Mecking S. (2023). Degradable and Recyclable
Polyesters from Multiple Chain Length Bio- and Waste-Sourceable Monomers. Angew. Chem., Int. Ed..

[17] Birkle M., Mehringer H. S., Nelson T. F., Mecking S. (2024). Aliphatic Polyester
Materials from Renewable 2,3-Butanediol. ACS
Sustainable Chem. Eng..

[18] Hester H. G., Abel B. A., Coates G. W. (2023). Ultra-High-Molecular-Weight
Poly­(Dioxolane):
Enhancing the Mechanical Performance of a Chemically Recyclable Polymer. J. Am. Chem. Soc..

[19] Zhao Y., Rettner E., Battson M., Hu Z., Miscall J., Rorrer N., Miyake G. (2025). Tailoring the Properties
of Chemically
Recyclable Polyethylene-Like Multiblock Polymers by Modulating the
Branch Structure. Angew. Chem. Int. Ed..

[20] Johnson A. M., Johnson J. A. (2023). Thermally Robust
yet Deconstructable and Chemically
Recyclable High-Density Polyethylene (HDPE)-Like Materials Based on
Si–O Bonds. Angew. Chem., Int. Ed..

[21] Zhao Y., Rettner E. M., Harry K. L., Hu Z., Miscall J., Rorrer N. A., Miyake G. M. (2023). Chemically recyclable
polyolefin-like
multiblock polymers. Science.

[22] Kocen A. L., Cui S., Lin T.-W., La Pointe A. M., Coates G. W. (2022). Chemically Recyclable
Ester-Linked Polypropylene. J. Am. Chem. Soc..

[23] Baur M., Mast N. K., Brahm J. P., Habé R., Morgen T. O., Mecking S. (2023). High-Density Polyethylene with In-Chain
Photolyzable and Hydrolyzable Groups Enabling Recycling and Degradation. Angew. Chem., Int. Ed..

[24] Ellis L. D., Rorrer N. A., Sullivan K. P., Otto M., McGeehan J. E., Román-Leshkov Y., Wierckx N., Beckham G. T. (2021). Chemical and biological
catalysis for plastics recycling and upcycling. Nat. Catal..

[25] Baur M., Lin F., Morgen T. O., Odenwald L., Mecking S. (2021). Polyethylene materials
with in-chain ketones from nonalternating catalytic copolymerization. Science.

[26] Pepels M. P. F., Hansen M. R., Goossens H., Duchateau R. (2013). From Polyethylene
to Polyester: Influence of Ester Groups on the Physical Properties. Macromolecules.

[27] Eck M., Mecking S. (2024). Closed-Loop Recyclable
and Nonpersistent Polyethylene-like
Polyesters. Acc. Chem. Res..

[28] Schwab S. T., Bühler L. Y., Schleheck D., Nelson T. F., Mecking S. (2024). Correlation
of Enzymatic Depolymerization Rates with the Structure of Polyethylene-Like
Long-Chain Aliphatic Polyesters. ACS Macro Lett..

[29] Eck M., Schwab S. T., Nelson T. F., Wurst K., Iberl S., Schleheck D., Link C., Battagliarin G., Mecking S. (2023). Biodegradable High-Density
Polyethylene-like Material. Angew. Chem., Int.
Ed..

[30] Ortmann P., Mecking S. (2013). Long-Spaced Aliphatic Polyesters. Macromolecules.

[31] Stempfle F., Ortmann P., Mecking S. (2016). Long-Chain
Aliphatic Polymers To
Bridge the Gap between Semicrystalline Polyolefins and Traditional
Polycondensates. Chem. Rev..

[32] Pepels M. P. F., Hofman W. P., Kleijnen R., Spoelstra A. B., Koning C. E., Goossens H., Duchateau R. (2015). Block Copolymers
of “PE-Like” Poly­(pentadecalactone) and Poly­(l-lactide):
Synthesis, Properties, and Compatibilization of Polyethylene/Poly­(l-lactide)
Blends. Macromolecules.

[33] Gazzano M., Malta V., Focarete M. L., Scandola M., Gross R. A. (2003). Crystal
structure of poly­(ω-pentadecalactone). J. Polym. Sci., Part B: Polym. Phys..

[34] Pascual A., Sardon H., Veloso A., Ruipérez F., Mecerreyes D. (2014). Organocatalyzed Synthesis of Aliphatic
Polyesters from
Ethylene Brassylate: A Cheap and Renewable Macrolactone. ACS Macro Lett..

[35] van
der Meulen I., Gubbels E., Huijser S., Sablong R., Koning C. E., Heise A., Duchateau R. (2011). Catalytic
Ring-Opening Polymerization of Renewable Macrolactones to High Molecular
Weight Polyethylene-like Polymers. Macromolecules.

[36] Stempfle F., Ortmann P., Mecking S. (2013). Which Polyesters
Can Mimic Polyethylene?. Macromol. Rapid Commun..

[37] Gaines T. W., Nakano T., Chujo Y., Trigg E. B., Winey K. I., Wagener K. B. (2015). Precise Sulfite Functionalization of Polyolefins via
ADMET Polymerization. ACS Macro Lett..

[38] Arrington A. S., Long T. E. (2022). Influence of carboxytelechelic oligomer molecular weight
on the properties of chain extended polyethylenes. Polymer.

[39] Han X.-W., Zhang X., Zhou Y., Maimaitiming A., Sun X.-L., Gao Y., Li P., Zhu B., Chen E. Y. X., Kuang X., Tang Y. (2024). Circular olefin
copolymers
made de novo from ethylene and α-olefins. Nat. Commun..

[40] Shiono T., Naga N., Soga K. (1991). Synthesis
of recyclable polyolefins
from α,ω-dihydroxypolybutadiene using condensation and
hydrogenation reactions. Macromol. Rapid Commun..

[41] Arroyave A., Cui S., Lopez J. C., Kocen A. L., La Pointe A. M., Delferro M., Coates G. W. (2022). Catalytic Chemical Recycling of Post-Consumer
Polyethylene. J. Am. Chem. Soc..

[42] Kobayashi S., Fukuda K., Kataoka M., Tanaka M. (2016). Regioselective Ring-Opening
Metathesis Polymerization of 3-Substituted Cyclooctenes with Ether
Side Chains. Macromolecules.

[43] Orski S. V., Kassekert L. A., Farrell W. S., Kenlaw G. A., Hillmyer M. A., Beers K. L. (2020). Design
and Characterization of Model Linear Low-Density
Polyethylenes (LLDPEs) by Multidetector Size Exclusion Chromatography. Macromolecules.

[44] Thompson C. B., Orski S. V. (2023). Synthesis and Dilute
Solution Properties of Precision
Short-Chain Branched Poly­(ethylene) Block Copolymers Derived from
Ring-Opening Metathesis Polymerization. Macromolecules.

[45] Sanchez I. C., Eby R. K. (1975). Thermodynamics and Crystallization of Random Copolymers. Macromolecules.

[46] Rojas G., Inci B., Wei Y., Wagener K. B. (2009). Precision Polyethylene:
Changes in Morphology as a Function of Alkyl Branch Size. J. Am. Chem. Soc..

[47] Marxsen S. F., Häußler M., Mecking S., Alamo R. G. (2021). Unlayered–Layered
Crystal Transition in Recyclable Long-Spaced Aliphatic Polyesters. ACS Appl. Polym. Mater..

[48] Jang Y.-J., Nguyen S., Hillmyer M. A. (2024). Chemically
Recyclable Linear and
Branched Polyethylenes Synthesized from Stoichiometrically Self-Balanced
Telechelic Polyethylenes. J. Am. Chem. Soc..

[49] Sworen J. C., Smith J. A., Berg J. M., Wagener K. B. (2004). Modeling Branched
Polyethylene: Copolymers Possessing Precisely Placed Ethyl Branches. J. Am. Chem. Soc..

[50] Arriola D. J., Carnahan E. M., Hustad P. D., Kuhlman R. L., Wenzel T. T. (2006). Catalytic
Production of Olefin Block Copolymers via Chain Shuttling Polymerization. Science.

[51] Guinier, A. X-ray diffraction in crystals, imperfect crystals, and amorphous bodies; WH Freeman and Company, 1963.

[52] Butler M. F., Donald A. M., Bras W., Mant G. R., Derbyshire G. E., Ryan A. J. (1995). A Real-Time Simultaneous Small- and
Wide-Angle X-ray
Scattering Study of In-Situ Deformation of Isotropic Polyethylene. Macromolecules.

[53] Stribeck, N. X-Ray Scattering of Soft Matter; Springer Nature, 2007.

[54] Starck P. (1997). Dynamic Mechanical
Thermal Analysis On Ziegler-Natta And Metallocene Type Ethylene Copolymers. Eur. Polym. J..

[55] Sirotkin R. O., Brooks N. W. (2001). The dynamic mechanical
relaxation behaviour of polyethylene
copolymers cast from solution. Polymer.

[56] Wang H. P., Khariwala D. U., Cheung W., Chum S. P., Hiltner A., Baer E. (2007). Characterization of Some New Olefinic Block Copolymers. Macromolecules.

[57] Bensason S., Stepanov E. V., Chum S., Hiltner A., Baer E. (1997). Deformation
of Elastomeric Ethylene–Octene Copolymers. Macromolecules.

[58] Kruszynski J., Nowicka W., Bouyahyi M., Liu Y., Yang L., Rozanski A., Anbuchezhian N., Jasinska-Walc L., Duchateau R. (2023). Unprecedented Adhesive Performance
of Propylene-Based
Hydroxyl-Functionalized Terpolymers. ACS Appl.
Polym. Mater..

[59] Nakao K. (1972). Relationship
Between Bond Strength and Crystallinity of High Polymers-Polyethylene,
Polyethyleneterephthalate, and Nylon. J. Adhes..

[60] Sun H. (1993). Ab initio
characterizations of molecular structures, conformation energies,
and hydrogen-bonding properties for polyurethane hard segments. Macromolecules.

[61] Guo Y., Liu J., Lu Y., Dong D., Wang W., Zhang L. (2018). A combined
molecular dynamics simulation and experimental method to study the
compatibility between elastomers and resins. RSC Adv..

[62] Gupta J., Nunes C., Vyas S., Jonnalagadda S. (2011). Prediction
of Solubility Parameters and Miscibility of Pharmaceutical Compounds
by Molecular Dynamics Simulations. J. Phys.
Chem. B.

[63] Kruszynski J., Nowicka W., Pasha F. A., Yang L., Rozanski A., Bouyahyi M., Kleppinger R., Jasinska-Walc L., Duchateau R. (2025). Tuning the Adhesive Strength of Functionalized Polyolefin-Based
Hot Melt Adhesives: Unexpected Results Leading to New Opportunities. Macromolecules.

