# Supplementary material for: Polyethylene-like Polyesters: Strategies for Tailoring Mechanical Properties and Adhesion Performance
Source: Macromolecules. 2025 Aug 22;58(17):9440–9. doi: 10.1021/acs.macromol.5c01495 (PMC12424291; doi:10.1021/acs.macromol.5c01495)
Supplement: Supplementary file 1 [file ma5c01495_si_001.pdf]

Supporting Information

Polyethylene-like Polyesters: Strategies for Tailoring Mechanical Properties and Adhesion Performance

Weronika Nowicka,[a,b] Artur Rozanski,[c] Farhan Ahmad Pasha,[d] Lidia Jasinska-Walc,[a,b] Rob Duchateau*[e,b]*

a) Department of Chemistry and Technology of Functional Materials, Chemical Faculty, Gdansk University of Technology, G. Narutowicza Str. 11/12, 80-233 Gdansk, Poland.

b) SABIC Technology & Innovation, Urmonderbaan 22, 6167 RD Geleen, The Netherlands.

c) Centre of Molecular and Macromolecular Studies, Polish Academy of Sciences, Sienkiewicza 112, 90-363 Lodz, Poland.

d) SABIC Technology Center at KAUST, 25 Unity Blvd, Thuwal 23955, Saudi Arabia.

e) Chemical Product Engineering, Department of Chemical Engineering, University of Groningen, Nijenborgh 4, 9747 AG Groningen, The Netherlands.

Additional experimental details, materials, and methods

General Considerations. All experiments were performed under an inert dry nitrogen atmosphere using either standard Schlenk or glove box techniques. *Cis*-cyclooctene and *cis*-1,4-diacetoxy-2-butene (CTA) were purchased from Sigma-Aldrich, distilled prior to use, and kept under inert

atmosphere. The 2nd generation Grubbs catalyst (M204), sodium ethoxide (21 wt% in ethanol), tetrahydrofuran (> 99%, anhydrous), HCl (37%), tin(II) 2-ethylhexanoate (92.5% – 100%) and succinic anhydride were purchased from Sigma Aldrich and used without purification. Toluene was purified using an mBraun MB-SPS solvent purification system and kept under a nitrogen atmosphere. Methanol was purchased from Thermo Scientific and used without purification. Hydrogenated α,ω-dihydroxy polybutadiene (soft block, Krasol® HLBH P-3000) was purchased from Cray Valley Total Energies and used as such for polycondensations. Antioxidant (Irganox 1010) was purchased from BASF and used as such. HDPE (B6246LS) and mLLDPE (SUPEER 8115) were provided by from SABIC. Aluminum (A35, mill finish, Q-Panel) was employed as surface for adhesive experiments.

Analytical Techniques

M_n, M_w and the polydispersity index ($Đ_M$) were determined using Size Exclusion Chromatography (SEC). The measurements were performed at 150 ˚C on a Polymer Char GPC-IR built around an Agilent GC oven model 7890B, equipped with guard column (PLgel Olexis 50×7.5 mm), 3 columns (PLgel Olexis 300×7.5 mm), an autosampler and the Integrated Detector IR4. 1, 2-dichlorobenzene (*o*DCB) was used as an eluent at a flow rate of 1 mL/min. Measurements of average number of short chain branches per 1000 carbons in total was performed at 150 ˚C on a Polymer Char GPC-IR built around an Agilent GC oven model 7890B, equipped with guard column (PLgel Olexis 50×7.5 mm), 3 columns (PLgel Olexis 300×7.5 mm), an autosampler and the Integrated Detector IR5. 1, 2, 3-trichlorobenzene (TCB) was used as an eluent at a flow rate of 1 mL/min. Mark-Houwink parameters for polyethylene, where $K = 4.416 \times 10^{-4}$ (dL/g) and $\alpha = 0.725$, were used. Polystyrene (PS) standards were selected to provide calibration points. The SEC data were processed using Calculations Software GPC One.

The degree of polymerization (P_n) was calulated using M_n determined by SEC. P_n is defined as:

$$P_{\mathrm{n}} = \frac{M_{\mathrm{n}(PE-SB/HB)}}{\left(X_{SB} * M_{\mathrm{n}(SB)} + X_{HB} * M_{\mathrm{n}(HB)}\right)}, where\ M_{\mathrm{n(SB)}} = 2.0\,\frac{\mathrm{kg}}{\mathrm{mol}};\ M_{\mathrm{n}(HB)} = 2.9\,\frac{\mathrm{kg}}{\mathrm{mol}}$$

Melting (T_{m}) and crystallization (T_{c}) temperatures, as well as enthalpies of the thermal transitions, were measured by DSC using a DSC Q100 from TA Instruments. The measurements were carried out at a heating and cooling rate of 10 °C/min from −40 °C to 230 °C. The transitions were deduced from the second heating and cooling curves.

Density was determined using hydrostatic balance with Sartorius Density Kit YDK04 at room temperature in water. The reported values are an average of at least 5 measurements.

^{1}H NMR analysis was carried out at 125 °C using deuterated tetrachloroethane (TCE-D2) as solvent, butylated hydroxytoluene (BHT) as antioxidant and recorded using 5 mm tubes on a Bruker Avance500 spectrometer operating at a frequency of 400 MHz. Chemical shifts are reported in ppm versus tetramethylsilane (TMS, 0.00 ppm) and were determined by reference to the residual solvent protons.

Dynamic mechanical thermal analysis (DMTA) was performed using a TA Instruments Q800 DMA. Samples were tested by strain-controlled temperature ramp with the frequency of 1 Hz. The measurements were performed using film-tension mode. The applied temperature profile was from −150 °C to 150 °C with the ramp 2 °C/min. The glass transition temperature was calculated as the maximum of the tangent delta signal.

Analysis of the crystalline structure of the materials was performed using wide-angle X-ray scattering (WAXS) measurements by means of a computer-controlled goniometer coupled to a sealed-tube source of Cu Kα radiation (Philips), operating at 50 kV and 30 mA. The Cu Kα line was filtered using electronic filtering and the usual thin Ni filter.

The Kiessig-type camera with sample detector distance of 1.2 m was coupled to an X-ray CuK a low divergence microsource, operating at 50 kV and 1 mA (GeniX Cu-LD by Xenocs, France). The small angle X-ray scattering (SAXS) produced by the sample was recorded with the

Pilatus 100 K solid-state area detector of the resolution of 172 × 172 μm^2 (Dectris, Switzerland). Dimension of scattering objects was determined from one dimensional sections of 2-D pattern. Background and Lorentz corrections were applied to the curves. Dimension of scattering objects was then calculated from position of the maximum of corrected curves using the Braggs law.

Tensile tests were performed using a Zwick type Z020 tensile tester equipped with a 2.5 kN load cell according to ISO 527-3 standard (specimen type B5). A grip-to-grip separation of 15 mm was used. The samples were pre-stressed to 3 N, then loaded with a constant crosshead speed 15 mm/min. The integration of stress versus extension graphs for determination of polymers' toughness were achieved using the Integrate feature from OriginPro software, version 2018. The reported values are an average of at least 5 measurements.

The laminated samples, used for the lap shear test, were prepared via compression-molding using LabEcon 600 high-temperature press (Fontijne Presses, the Netherlands). Namely, the films (25 mm × 12.5 mm × 0.5 mm) of olefin block copolymer mimics were loaded between the substrates: Steel QD35, Aluminum A35 with overlap of 12.5 mm (312.5 mm^2 bonding area). Then, the compression-molding cycle was applied: (i) heating to 180 ˚C, (ii) stabilizing for 5 min without force applied, (iii) for 5 min with 100 kN (2 MPa) normal force and cooling down to 40 ˚C with the cooling speed of 10 ˚C/min under 100 kN (0.6 MPa) normal force.

Lap Shear Strength test (LSS) measurements – performed following the ISO 4587 procedure – were carried out using Zwick type Z020 tensile tester equipped with a 10 kN load cell. The tests were performed on standardized specimens (10 cm × 2.5 cm) with surface overlapping 12.5 mm under room temperature. A grip-to-grip separation of 140 mm was used. The samples were pre-stressed to 3 N, then loaded with a constant crosshead speed 100 mm/min. To calculate the lap shear strength, the reported force value divided by the bonding surface (312.5 mm^2) of the specimens. The reported values are an average of at least 5 measurements of each composition.

Adhesion is determined as the maximum force (N) of the adhesive connection divided by the overlap area of the specimen A (mm^2). Work of debonding is defined as:

$$Work\ of\ debonding\ \left(\frac{\mathrm{N}}{\mathrm{m}}\right) = \frac{\int_0^x F\,dx}{A}$$

The integration of force versus extension graph $\int_0^x F\,dx$ was achieved using the Integrate feature from OriginPro software, version 2018.[1, 2]

Synthesis of α,ω-dihydroxy poly(*cis*-cyclooctene).

Reactions were performed using standard Schlenk techniques. *cis*-Cyclooctene (COC) (50.0 g, 0.45 mol), 1,4-bis(acetoxy)-2-butene (CTA) (3.9 g, 22 mmol) and the 2nd generation ruthenium Grubbs catalyst (14.4 mg, 23 µmol) dissolved in a minimal amount of toluene were transferred into the flask and secured with a stopper. Then the flask was placed in an oil-bath preheated to 50 ˚C. After 24 h, the reaction was quenched by transferring the reaction mixture into acidified methanol (0.1M HCl). After stirring for 1 h to remove catalyst residues, the crude product was dissolved in toluene (20 mL) by heating to 100 ˚C and subsequently precipitated in a large excess (200 mL) of slightly acidified cold methanol. The mixture was allowed to warm to room temperature and the precipitated polymer was isolated by filtration, followed by washing with methanol. The filtrated methanol solution colored yellow. The purification step was repeated until the methanol was colorless and the polymer product a white solid. Subsequently, the dried polymer was dissolved in a mixture of toluene (100 mL) and THF (300 mL) at 80 ˚C under a nitrogen atmosphere. The thus obtained polymer solution was cooled by placing it in ice bath and EtONa (200 mL, 0.5 M) was gradually added after which the mixture was left stirring overnight. The resulting yellow mixture was poured into acidified methanol (0.1 M HCl) and subsequently filtered to give α,ω-dihydroxy poly(*cis*-cyclooctene) as a white solid. The material was then hydrogenated

using standard procedure with *p*-toluenesulfonohydrazide with tripropyloamine in refluxing xylenes as described in the literature.[3]

Synthesis of olefin block copolymers mimics.

Hydrogenated α,ω-dihydroxy poly(*cis*-cyclooctene) (3 g, 1.1 mmol), succinic anhydride (414 mg, 4.2 mmol), tin 2-ethylhexanoate (8.4 mg, 20.7 μmol, 1 mol% catalyst solution in toluene were prepared in a glove box under an inert dry nitrogen atmosphere) and Irganox 1010 (1.0 M, 500 ppm) were introduced into the reactor that was subsequently degassed 5 times with nitrogen purge and vacuum cycles. The polymerization mixture was first heated to 190 ˚C under constant stirring (200 rpm) and under a nitrogen atmosphere. The initial esterification reaction was carried out for 4.5 h at atmospheric pressure. Then the temperature was increased to 200 ˚C for 30 min and to 210 ˚C for another 30 minutes. Subsequently, the polycondensation was performed by gradual elevation of the temperature until 230 ˚C, after which vacuum was applied (0.5 – 1.0 mbar) and the reaction was conducted for another 30 min. Then, the polymer was collected as a melt and cooled to room temperature. Series of olefin block copolymers mimics were synthesized with the molar ratio of hydrogenated α,ω-dihydroxy polybutadiene: hydrogenated α,ω-dihydroxy poly(*cis*-cyclooctene):succinic anhydride equal to: 0:1:2, 0.2:0.8:2, 0.4:0.6:2, 0.6:0.4:2, 0.8:0.2:2 and 1:0:2.

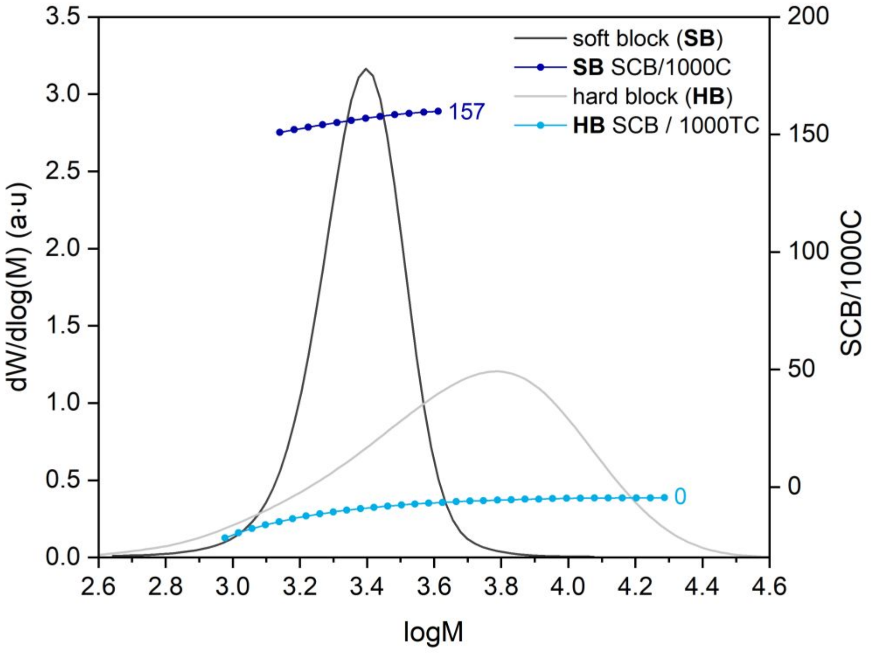

Figure S1. HT-SEC profile of soft block (SB) and hard block (HB) and average number of short chain branching per 1000 carbons (SCB/1000C).

Determination of average number of short chain branching (SCB/1000C) using ^{1}H NMR.

Average number of SCB/1000C for PE-like polyesters was determined using the following equation. Integrals were calculated based on Figure S1 given as an example.

$$\frac{\mathrm{SCB}}{1000\mathrm{C}}(\mathrm{NMR}) = \frac{\frac{I_1}{3}}{\frac{I_1}{3} + \frac{\frac{I_1}{3} + I_2}{2} + \frac{I_3}{4} + \frac{I_4}{4} + \frac{I_5}{4} + \frac{I_6}{4}} \times 1000$$

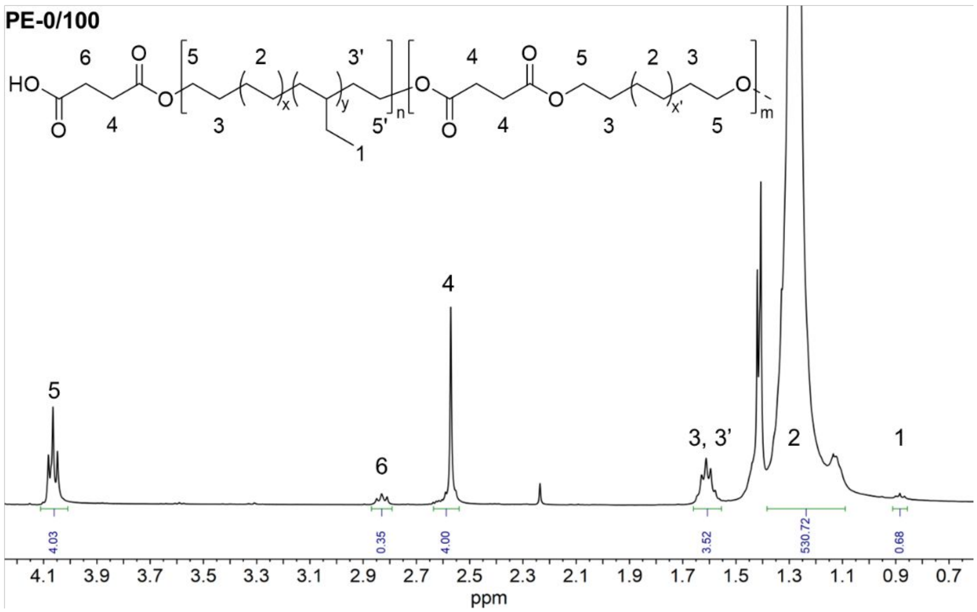
PE-0/100
5
4
6
3, 3'
2
1
4.03
0.35
4.00
3.52
530.72
0.68
4.1 3.9 3.7 3.5 3.3 3.1 2.9 2.7 2.5 2.3 2.1 1.9 1.7 1.5 1.3 1.1 0.9 0.7
ppm

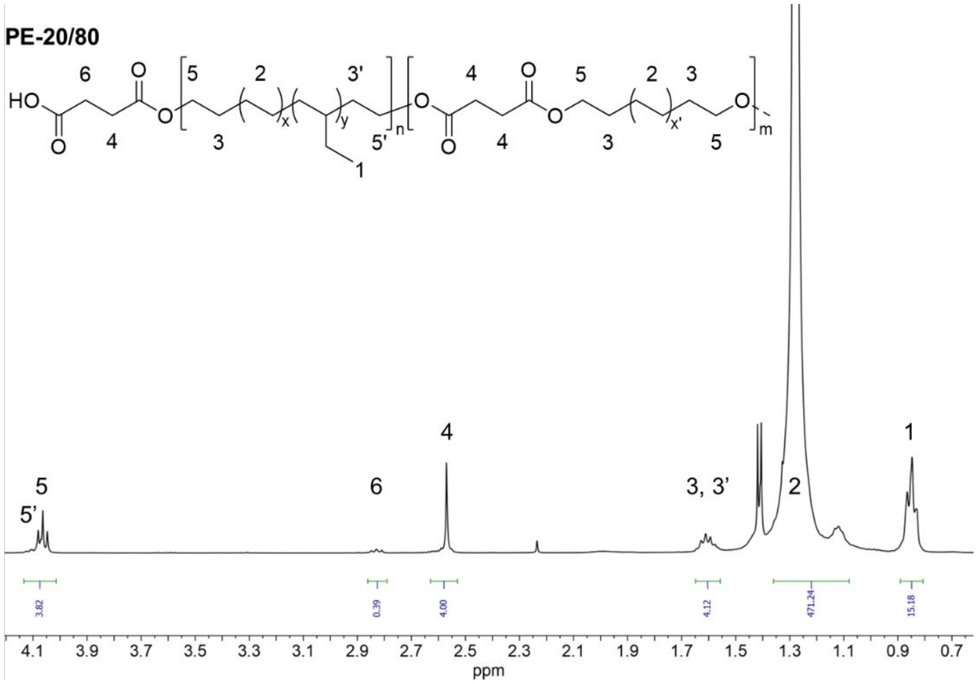
PE-20/80
5
5'
6
4
3, 3'
2
1
3.82
0.39
4.00
4.12
471.24
15.18
4.1 3.9 3.7 3.5 3.3 3.1 2.9 2.7 2.5 2.3 2.1 1.9 1.7 1.5 1.3 1.1 0.9 0.7
ppm

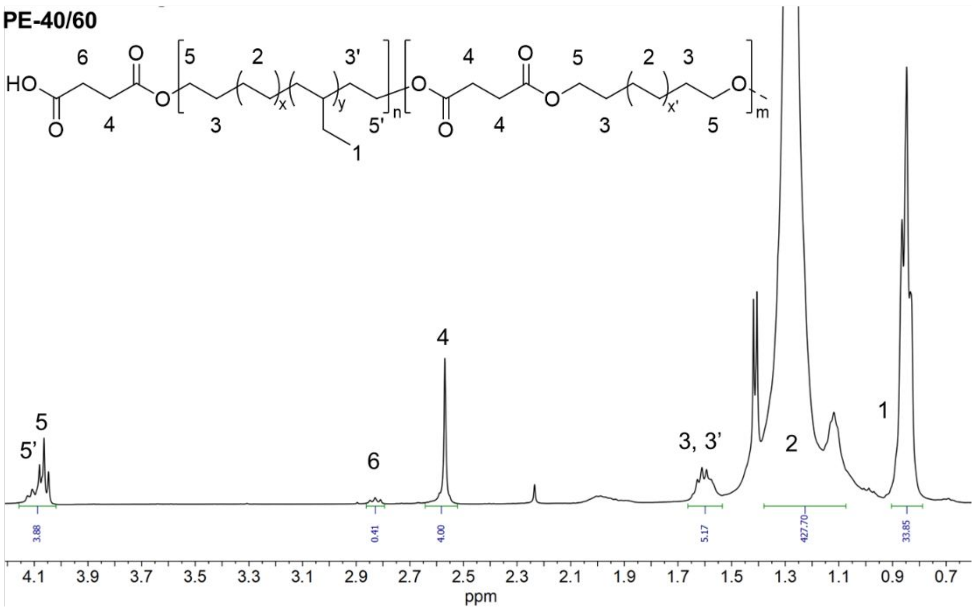
PE-40/60
HO
6
4
5
2
3
x
y
3'
5'
n
1
4
5
2
3
x'
5
m
5
5'
6
4
3, 3'
2
1
3.88
0.41
4.00
5.17
427.70
33.85
4.1 3.9 3.7 3.5 3.3 3.1 2.9 2.7 2.5 2.3 2.1 1.9 1.7 1.5 1.3 1.1 0.9 0.7
ppm

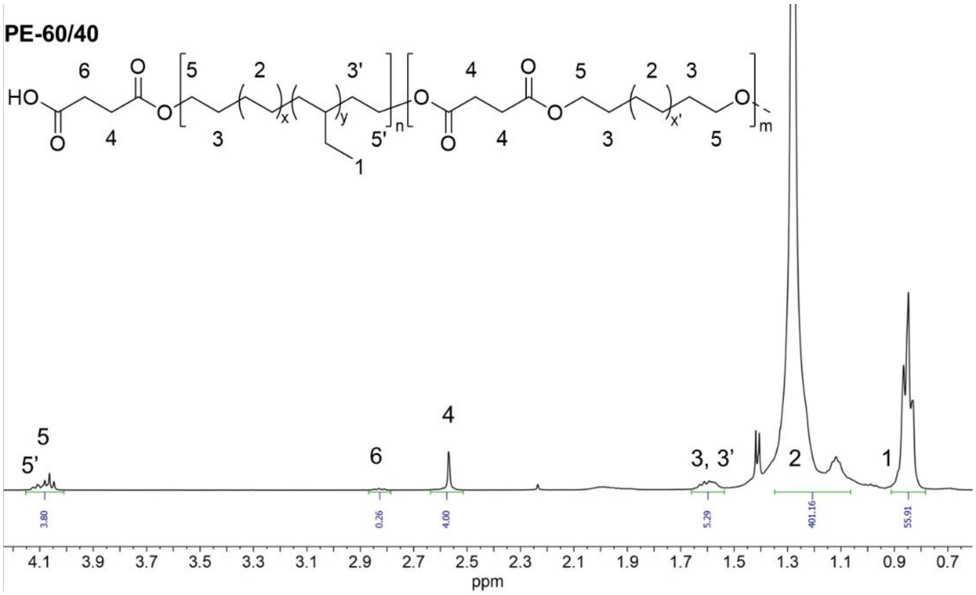
PE-60/40
HO
6
4
5
2
3
x
y
3'
5'
n
1
4
5
2
3
x'
5
m
5
5'
6
4
3, 3'
2
1
3.80
0.26
4.00
5.29
401.16
55.91
4.1 3.9 3.7 3.5 3.3 3.1 2.9 2.7 2.5 2.3 2.1 1.9 1.7 1.5 1.3 1.1 0.9 0.7
ppm

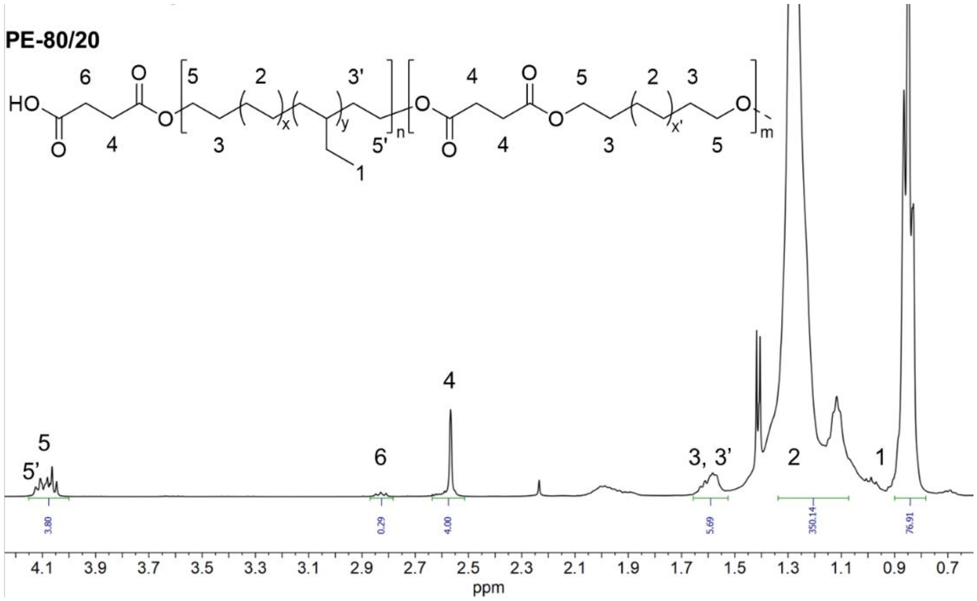

Figure S2. ^{1}H NMR spectra of PE-like polyesters (Table 1) used for the determination of SCB/1000C.

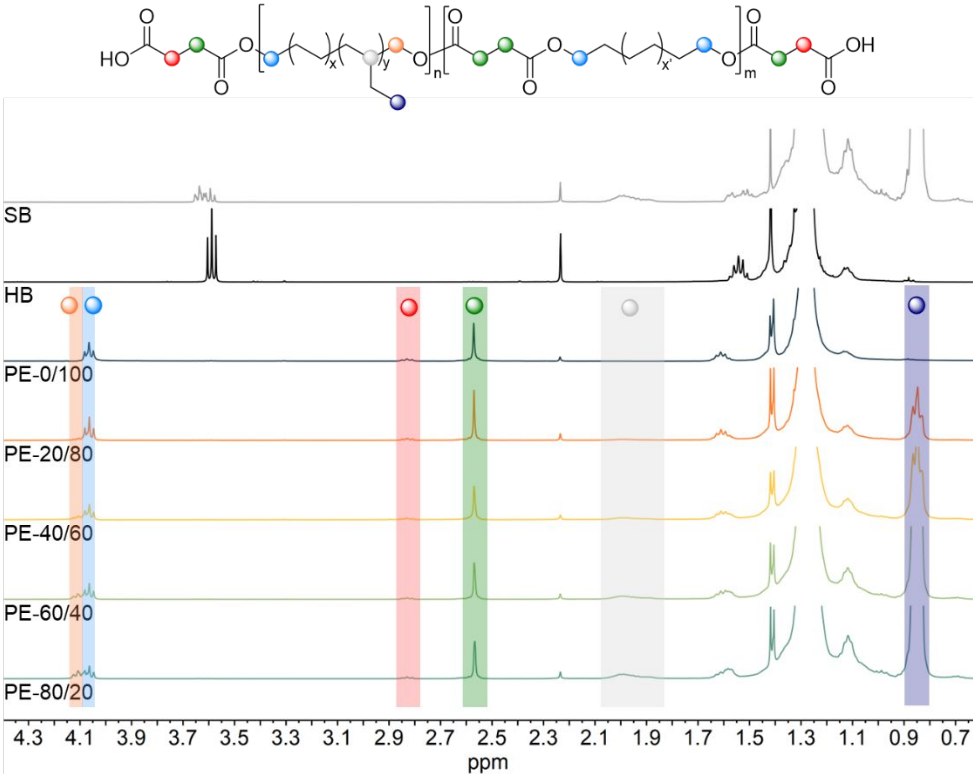

Figure S3. Example of ^{1}H NMR spectra of soft block (**SB**), hard block (**HB**) and PE-like polyesters.

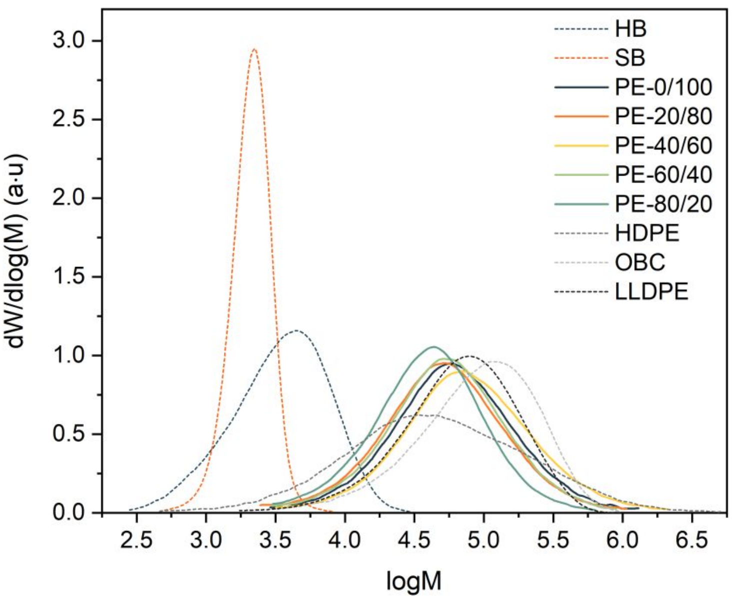

Figure S4. HT-SEC profiles of soft block (**SB**), hard block (**HB**), PE-like polyesters and **HDPE**, **LLDPE** and **OBC** reference samples.

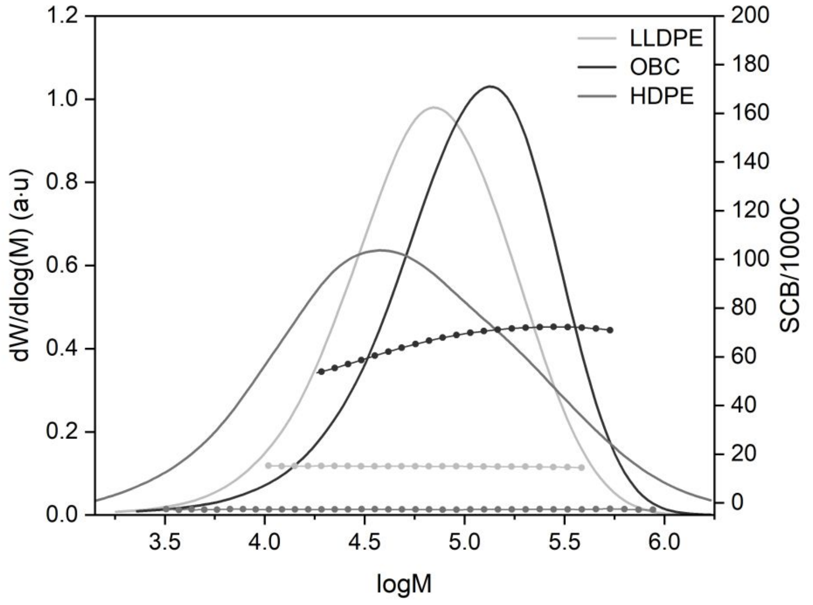

Figure S5. HT-SEC SCB profiles of **HDPE**, **LLDPE** and **OBC** reference samples.

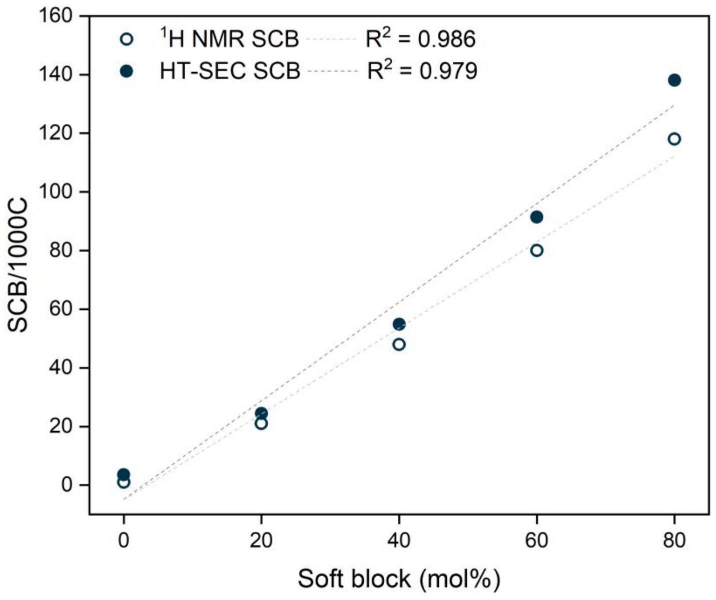

Figure S6. Linear correlation between soft block content and SCB/1000C values determined with HT-SEC and ^{1}H NMR.

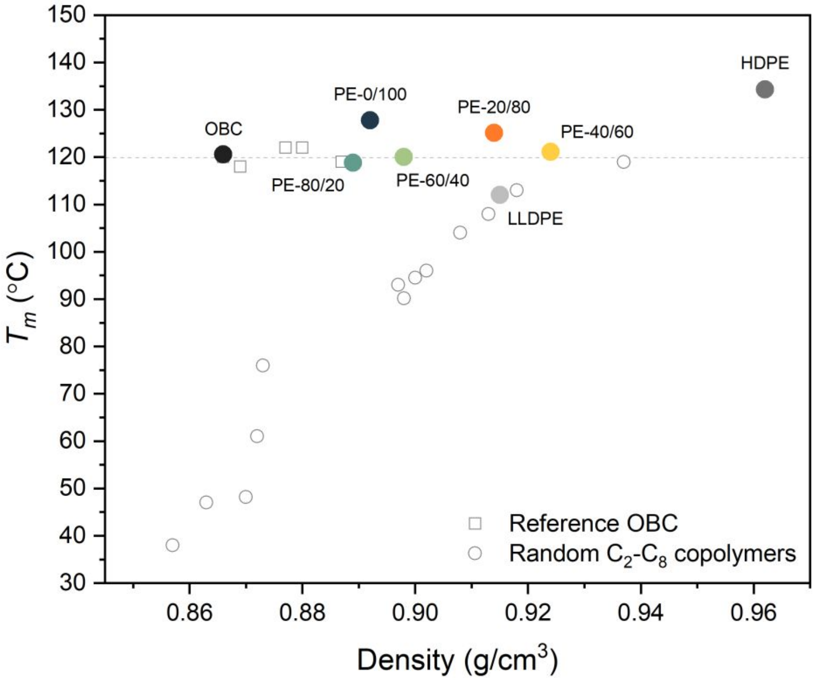

Figure S7. Melting point versus density of PE-like polyesters and **HDPE**, **LLDPE**, **OBC** reference samples and other reported random C_2-C_8 copolymers.[4]

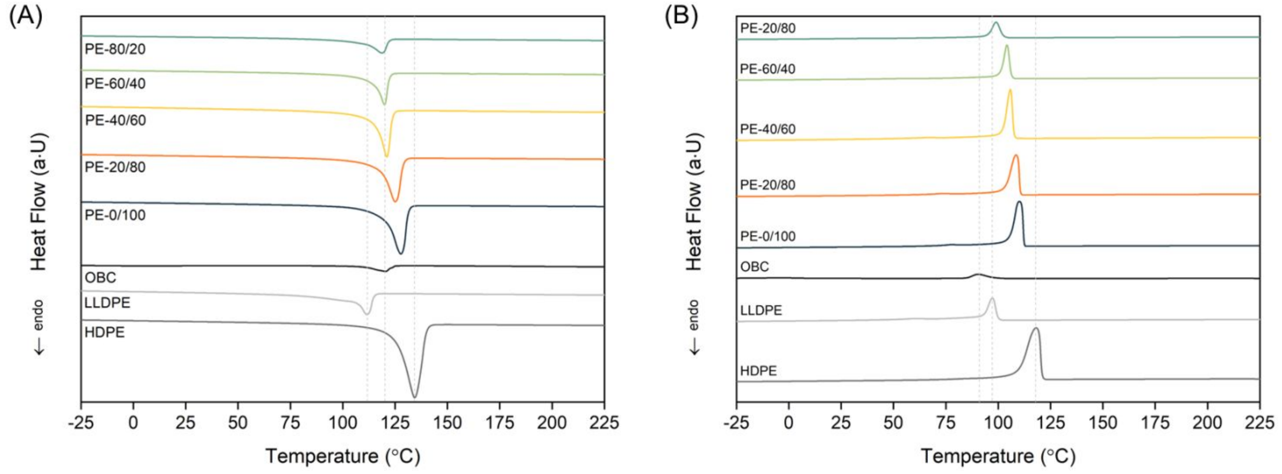

Figure S8. Melting (A) and crystallization (B) profiles of PE-like polyesters and reference polyolefins determined by DSC.

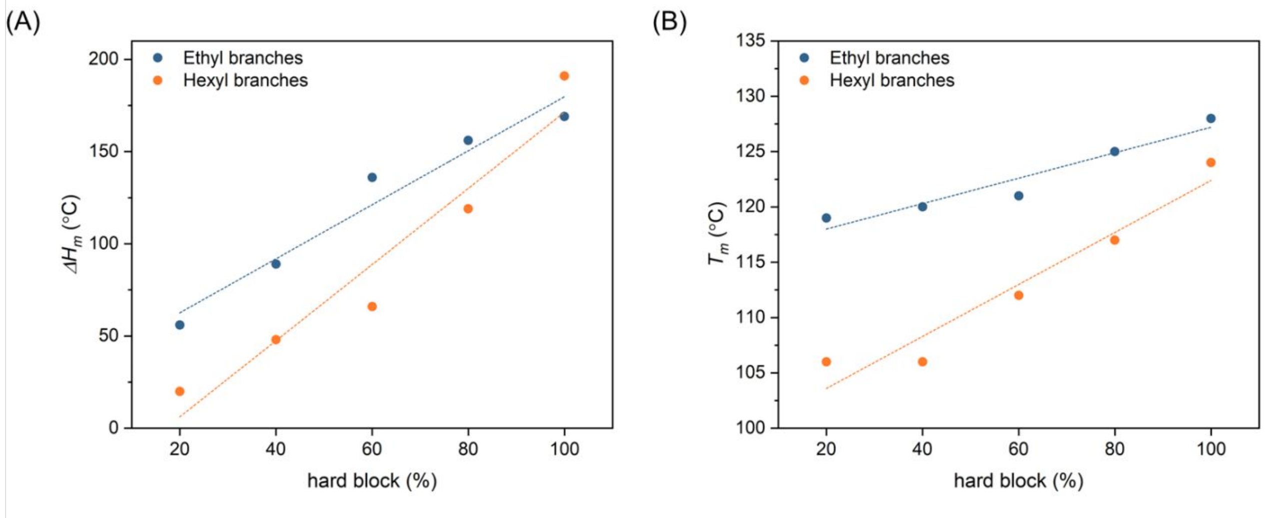

Figure S9. Comparison of the effect of the OBC soft/hard block composition on the melting enthalpy (A) and the melting temperature (B).

Table S1. Long period values, crystallinity level and crystal thickness determined with small angle X-ray scattering (SAXS) for series of PE-like polyesters and reference polyolefins.

Sample	T_m (˚C)	$\Delta H_m{}^a$ (J/g)	$X_c{}^a$ (%)	Long period LP (nm)	Crystal thickness (nm)
**PE-0/100**	127.8	169.2	57.7	25.8	16.5
**PE-20/80**	125.1	156.4	53.4	25.3	15.7
**PE-40/60**	121.1	136.3	46.5	25.3	14.9
**PE-60/40**	120.0	89.2	30.4	29.0	14.9
**PE-80/20**	118.8	56.4	19.2	36.5	14.2
**OBC**	120.6	21.0	7.2	29.4	5.8
**LLDPE**	112.0	99.6	34.0	14.8	8.4
**HDPE**	134.3	264.7	90.3	28.4	20.2

[a]Determined with DSC, where enthalpy of melting for pure polyethylene is $\Delta H_{m(PE)}$ = 293 J/g.

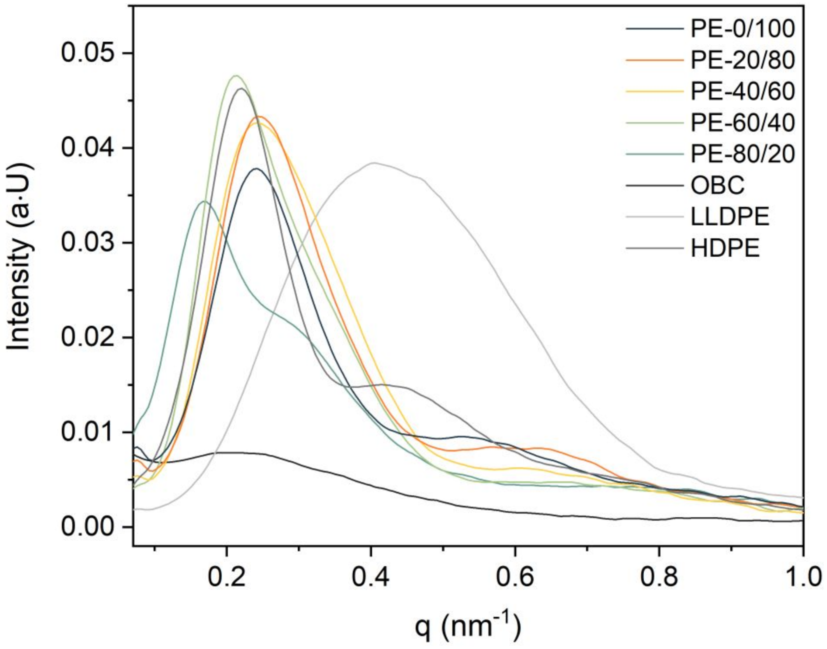

Figure S10. Small Angle X-Ray Scattering (SAXS) profiles of PE-like polyesters and polyolefin references.

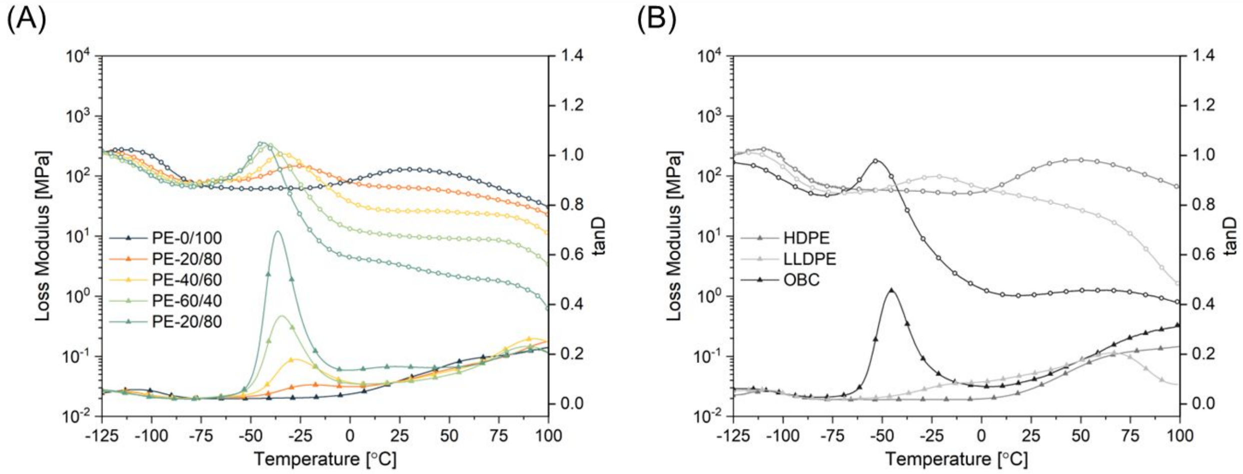

Figure S11. Loss modulus and tanδ for PE-like polyesters (A) and reference polyolefins (B).

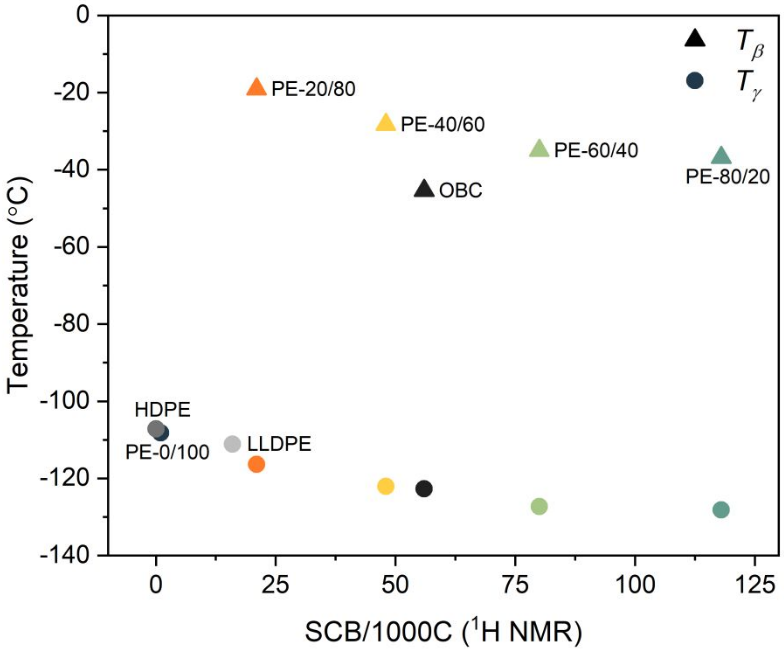

Figure S12. The correlation between T_γ, T_β and short chain branching per 1000C determined with ^{1}H NMR.

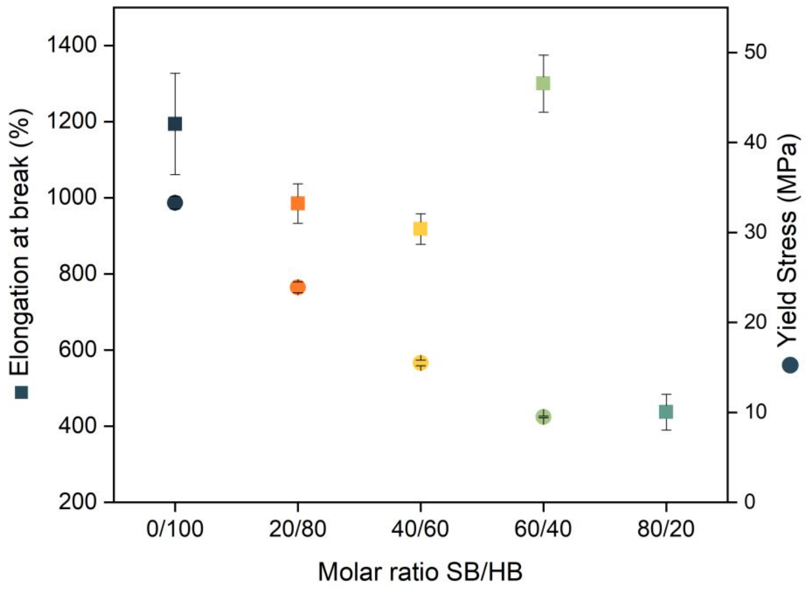

Figure S13. Elongation at break and yield stress versus composition of PE-like polyesters.

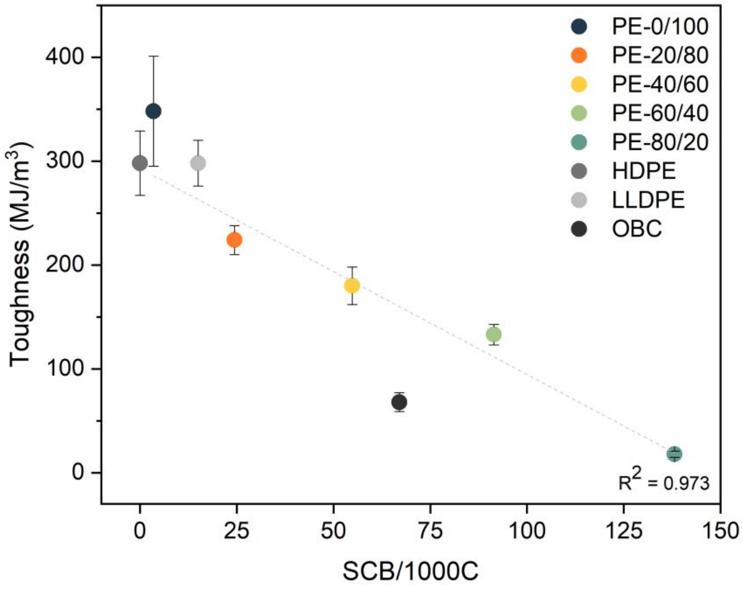

Figure S14. Correlation between branching content and toughness of PE-like polyesters. Polyolefin samples are added as references.

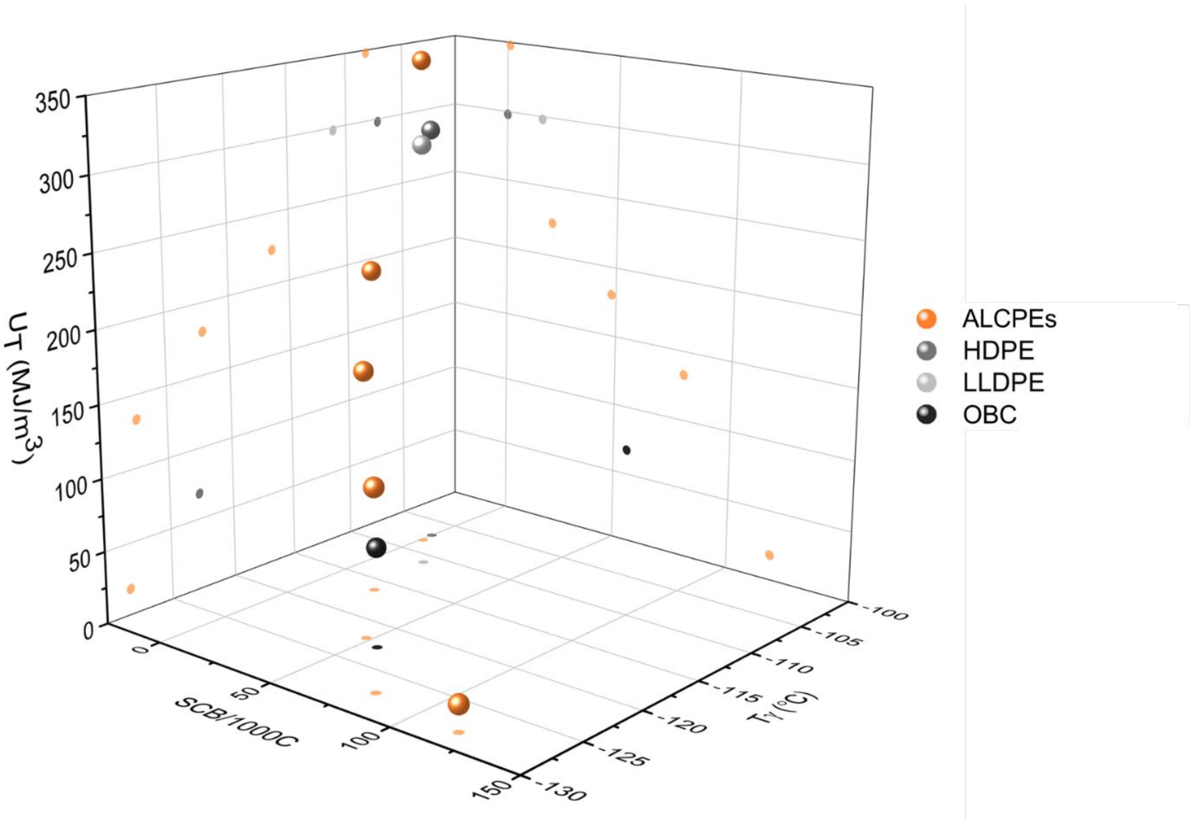

Figure S15. Correlation between the short chain branching (SCB) level, toughness (U_T) and low temperature glass transition (T_γ).

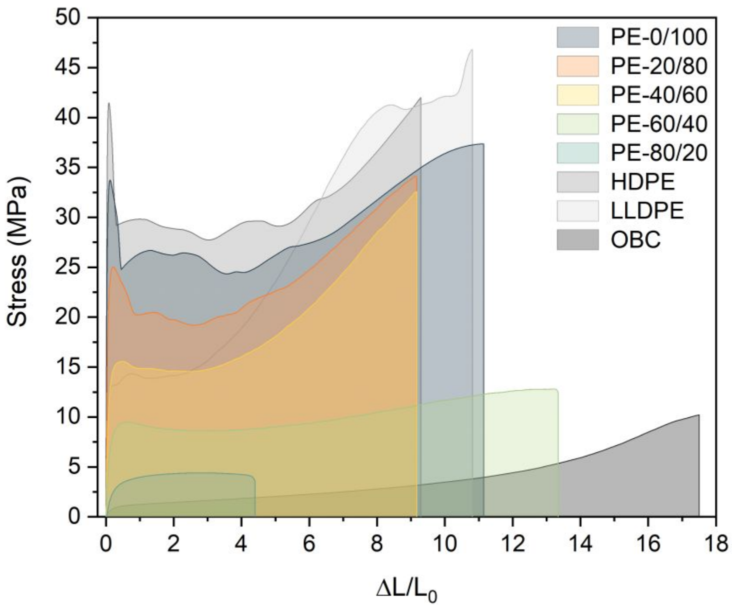

Figure S16. Graphic representation of toughness of LSAPEs and benchmark polyolefins, respectively, determined as the area under stress-strain plot.

Molecular Dynamics Modeling (MD)

Model preparation: The molecular chain of model **PE-0/100** ($\overline{M}_{n(MD\ PE\text{-}0/100)}$ = 38.73 kg/mol) was constructed using 13 units of the hard block, model **PE-40/60** ($\overline{M}_{n(MD\ PE\text{-}40/60)}$ = 43.66 kg/mol) was constructed using 8 units of the soft block and 9 units of the hard block, while model **PE-80/20** ($\overline{M}_{n(MD\ PE\text{-}40/60)}$ = 27.03 kg/mol) was constructed using 10 units of the soft block and 2 units of the hard block. The simulations were performed using BIOVIA Materials Studio 2022.[5] The geometry of each polymeric chain was optimized in 3D cubic cell using Amorphous Cell module.[6] Then the cubic cells were refined by ultra-short molecular dynamics NVT (constant number of particles, N, volume, V, and temperature, T) at process temperature (190 ˚C) with a time step of 0.5 fs for 5000 steps followed by an NPT ensemble run at 190 ˚C with a time step of 0.5 fs. Minimum fluctuations

in density and energy were two cut off criteria to determine the equilibrium of the system. Finally, the equilibrated cells were subjected to adhesion study. These simulations were performed using the Forcite module coupled with Condensed-Phase Optimized Molecular Potentials for Atomistic Simulations Studies (COMPASSIII) force field, which was selected for periodic boundary condition-based molecular dynamics calculations.[7-10]

Adhesion modelling: The Al_2O_3 crystal structure was taken from the BIOVIA library, and a surface (1 1 1) was created using 5 × thickness. The resulting surface was transformed into an 8 × 5 supercell consisting of 6000 atoms with lattice parameters (65.942638 × 69.176254 Å) having sufficient surface area to graft the sample size of the polymeric blend. Because there is no covalent bond in the metal oxide layer between the aluminum and oxide atoms, the aluminum and oxygen atoms in Al_2O_3 were set to constraints to avoid any disruption during minimization and simulation. The surface geometry was minimized using the COMPASSIII force field method. Furthermore, each polymeric model obtained at the end of molecular dynamics simulation was used to place over the Al_2O_3 surface. The final simulation box was built using the layer module along with 40 Å vacuum to break the periodic boundary conditions. The geometry optimization of the layer was carried out using the COMPASSIII force field method. Then annealing was carried out for 2 cycles with NVT Ensemble, and a short-term molecular dynamic 50 ps was performed using NVT Ensemble. Furthermore, three energy calculations were performed: surface-polymer system, surface alone, and polymer alone using the same box size, and the adhesion energy was calculated using equation presented below.

$$\Delta E_{\text{adh}} = E_{\text{Surface-Polymer}} - (E_{\text{Surface}} + E_{\text{Polymer}})$$

Table S2. Composition of polymeric models and Adhesion Energy (E_{adh}) over Al_2O_3 (111) surface.

Model	$M_{n(MD)}$ (SB/ HB) kg/mol	SB Number	HB Number	SB (wt%)	HB (wt%)	$M_{n(MD)}$ kg/mol	Adhesion Energy E_{adh} (kcal·mol^{-1})
**PE-0/100**	2.03/2.98	0	13	0%	100%	38.73	–1922.3
**PE-40/60**	2.03/2.98	8	9	40%	60%	43.66	–1724.8
**PE-80/20**	2.03/2.98	10	2	80%	20%	27.03	–1180.2

MD – Molecular Dynamics; SB – soft block; HB – hard block.

Bibliography:

(1) Rahman, M. A.; Bowland, C.; Ge, S.; Acharya, S. R.; Kim, S.; Cooper, V. R.; Chen, X. C.; Irle, S.; Sokolov, A. P.; Savara, A.; et al. Design of tough adhesive from commodity thermoplastics through dynamic crosslinking. *Sci. Adv.* **2021**, *7* (42), eabk2451.

(2) Mazzotta, M. G.; Putnam, A. A.; North, M. A.; Wilker, J. J. Weak Bonds in a Biomimetic Adhesive Enhance Toughness and Performance. *J. Am. Chem. Soc.* **2020**, *142* (10), 4762-4768.

(3) Fraser, C.; Hillmyer, M. A.; Gutierrez, E.; Grubbs, R. H. Degradable Cyclooctadiene/Acetal Copolymers: Versatile Precursors to 1,4-Hydroxytelechelic Polybutadiene and Hydroxytelechelic Polyethylene. *Macromolecules* **1995**, *28* (21), 7256-7261.

(4) Zhao, Y.; Rettner, E. M.; Harry, K. L.; Hu, Z.; Miscall, J.; Rorrer, N. A.; Miyake, G. M. Chemically recyclable polyolefin-like multiblock polymers. *Science* **2023**, *382* (6668), 310-314.

(5) BIOVIA MATERIALS: Studio Overview. Dassault Systemes, **2017**.

(6) Theodorou, D. N.; Suter, U. W. Detailed molecular structure of a vinyl polymer glass. *Macromolecules* **1985**, *18* (7), 1467-1478.

(7) Sun, H. Ab initio characterizations of molecular structures, conformation energies, and hydrogen-bonding properties for polyurethane hard segments. *Macromolecules* **1993**, *26* (22), 5924-5936.

(8) Sun, H. COMPASS: An ab Initio Force-Field Optimized for Condensed-Phase Applications Overview with Details on Alkane and Benzene Compounds. *J. Phys. Chem. B* **1998**, *102* (38), 7338-7364.

(9) McQuaid, M. J.; Sun, H.; Rigby, D. Development and validation of COMPASS force field parameters for molecules with aliphatic azide chains. *J. Comput. Chem.* **2004**, *25* (1), 61-71.

(10) Akkermans, R. L. C.; Spenley, N. A.; Robertson, S. H. COMPASS III: automated fitting workflows and extension to ionic liquids. *Mol. Simulat.* **2021**, *47* (7), 540-551.
